# Phosphatidylethanolamine homeostasis under conditions of impaired CDP-ethanolamine pathway or phosphatidylserine decarboxylation

**DOI:** 10.3389/fnut.2022.1094273

**Published:** 2023-01-05

**Authors:** Michaela St Germain, Roya Iraji, Marica Bakovic

**Affiliations:** Department of Human Health and Nutritional Science, College of Biological Sciences, University of Guelph, Guelph, ON, Canada

**Keywords:** serine, phospholipids, genetic disorders, ethanolamine, metabolism

## Abstract

Phosphatidylethanolamine is the major inner-membrane lipid in the plasma and mitochondrial membranes. It is synthesized in the endoplasmic reticulum from ethanolamine and diacylglycerol (DAG) by the CDP-ethanolamine pathway and from phosphatidylserine by decarboxylation in the mitochondria. Recently, multiple genetic disorders that impact these pathways have been identified, including hereditary spastic paraplegia 81 and 82, Liberfarb syndrome, and a new type of childhood-onset neurodegeneration-CONATOC. Individuals with these diseases suffer from multisystem disorders mainly affecting neuronal function. This indicates the importance of maintaining proper phospholipid homeostasis when major biosynthetic pathways are impaired. This study summarizes the current knowledge of phosphatidylethanolamine metabolism in order to identify areas of future research that might lead to the development of treatment options.

## Introduction

Phosphatidylethanolamine (PE) makes up 15–25% of the total lipids in the cell and plays a particularly important role in the inner leaflet of the plasma and mitochondrial membranes (1). It is composed of a glycerol backbone, linked to two fatty acid chains and a phosphoethanolamine head group. It is cone shaped and prefers a non-bilayer structure, thus inducing negative curvature when incorporated in cell membranes (2). The shape of PE also allows it to organize into the hexagonal phase, which contributes to the processes of cell fusion and fission (2, 3). The negative curvature that PE induces is also important for the maintenance of cristae in the inner mitochondrial membrane (IMM) (4). The cristae are important as they increase surface area and maximize ATP production (4).

There are two main pathways of PE synthesis in eukaryotic cells. In the endoplasmic reticulum (ER), PE is made by the CDP-ethanolamine (Kennedy) pathway. This pathway, often referred to as the *de novo* pathway of PE synthesis, converts exogenous ethanolamine (Etn) to PE through three enzymatic steps (5). In the mitochondria, PE is derived from phosphatidylserine by the decarboxylase (PSD) pathway. This pathway begins with the transport of phosphatidylserine (PS) into the mitochondria, where it is subsequently decarboxylated to form PE (6, 7). Although the CDP-Etn and PSD pathways both produce PE, a dysfunction in either pathway can be pathological (8–11). Dysfunction in the CDP-Etn branch of the Kennedy pathway has recently been linked to hereditary spastic paraplegia (OMIM #618768-Spastic paraplegia 81, autosomal recessive; SPG81 and OMIM#618770-Spastic paraplegia 82, autosomal recessive; SPG82) (8) and childhood-onset neurodegeneration named CONATOC (OMIM #618868—Neurodegeneration, childhood-onset, with ataxia, tremor, optic atrophy and cognitive decline; CONATOC) (9). Additionally, variations in the *PISD* gene, which codes for the PS decarboxylase enzyme (PSD) have been connected to a novel mitochondrial disease named Liberfarb syndrome (OMIM #618889–Liberfarb syndrome; LIBF) (10, 11). Since the major pathways of PE synthesis are unable to fully compensate for one another, it is critical to understand PE homeostasis and to find alternative methods of increasing PE levels when one pathway is dysfunctional.

Although PE metabolism and biosynthesis are well studied, there is no treatment for individuals who are deficient in PE. This study reviews the current knowledge of PE homeostasis to investigate potential mechanisms of increasing PE levels in the whole cell and mitochondria. The goal is to identify possible treatment options for individuals with genetic disorders that impact their ability to synthesize PE through either of the major pathways.

## Pathways of PE synthesis

**FIGURE 1:**
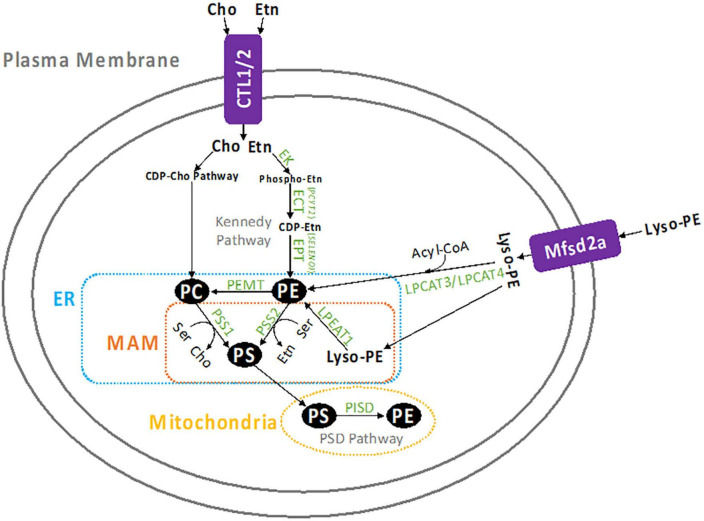
Summary of PE metabolism. Etn enters the cell by CTL1 or CTL2, where it is converted to PE by the CDP-Etn branch of the Kennedy pathway. First Etn is converted to phosphoethanolamine and then to CDP-Etn by ECT (*PCYT2*), which is the rate limiting step in this pathway. CDP-Etn is converted to PE by the EPT (*SELENOI*). Cho is similarly used to produce PC through the CDP-Cho branch of the Kennedy pathway. Once created, PE can be converted to PC by the PEMT enzyme (mostly in the liver), or to PS by PSS2 in the MAM. The PS can be transported to the mitochondria to be decarboxylated and form PE, which is catalyzed by the PISD enzyme. Similarly, PC can be converted to PS by PSS1 in the MAM, then decarboxylated by PISD in the mitochondria to form PE. Lyso-PE can also form PE when it is transported into the cell by the Mfsd2a transporter, the acylated by LPEAT1 in the MAM, or LPCAT2, or LPCAT3 in the cytosol.

There are four metabolic pathways that contribute to PE formation in the cell which are represented in Figure 1. The CDP-Etn Kennedy pathway and the PSD pathway are the two major producers of PE. Another pathway of PE production is the acylation of lyso-PE by acyl-CoA transferase enzymes, LPEAT1, LPCAT3, and LPCAT4 (12, 13). In humans, lyso-PE is likely transported into the cell by protein transporters which are not conclusively identified, however, there is evidence that Mfsd2a might be involved (14). In yeast, however, lyso-PE transport is known to be mediated by amino-phospholipid translocases Dnf1p and Dnf2p (12). Lyso-PE is also formed within the cell by hydrolysis of PE from the inner membrane (15). The acylation of lyso-PE takes place in the ER and requires acyl-CoA (12). The PE made in this pathway can access the same cellular compartments as PE made through the CDP-Etn pathway and can also supply PE to the mitochondria (4, 12). The fourth pathway of PE synthesis starts with the head group “base exchange” reactions between phosphatidylcholine (PC), PE and PS in the ER (16, 17). The head group base exchanges in PC (by PS synthase 1/PSS1/*PTDSS1*) and PE (by PS synthase 2/PSS2/*PTDSS2*) with serine (Ser) form PS and release free choline (Cho) and Etn. This is the only pathway to produce PS in mammalian cells and some of the newly made PS is transported and further decarboxylated to form PE in the mitochondria.

### The CDP-Etn Kennedy pathway

The CDP-Etn Kennedy pathway is the sole pathway for *de novo* synthesis of PE. This pathway involves the conversion of Etn and diacylglycerol (DAG) or alkyl-acyl-glycerol (AAG) to PE through three metabolic steps. In the first step, ethanolamine kinase (EK) converts Etn to phosphoethanolamine using ATP, leaving ADP as a by-product. Next, the CTP:phosphoethanolamine cytidyltransferase (ECT) catalyzes the formation of CDP-Etn by combining CTP and phosphoethanolamine and releasing pyrophosphate. This is the rate-limiting, and the most important regulatory step of the pathway (18). The final step is catalyzed by CDP-ethanolamine:1,2-diacylglycerol ethanolamine phosphotransferase (EPT). This enzyme forms PE from CDP-Etn and DAG (or AAG), and leaves cytidine monophosphate (CMP) as a by-product (19).

### Genes in the CDP-Etn Kennedy pathway

The genes involved in this pathway have all been identified and characterized. In humans, EK, ECT, and EPT are encoded by the genes ethanolamine kinase 1 and 2 (*ETNK1/ETNK2*), phosphate cytidylyltransferase 2 (*PCYT2*), and ethanolamine phosphotransferase 1 (*SELENO1*) or choline/ethanolamine phosphotransferase 1 (*CEPT1*) (19). Of these genes, *PCYT2* is of particular interest, as it is the sole gene that codes for ECT, which catalyzes the rate-limiting step in this pathway (18). This indicates that variations in *PCYT2* expression and activity can lead to specific dysfunction in the Kennedy pathway. Additionally, since the CDP-Etn pathway uses Etn as a substrate, the transport of Etn into the cell is also important. Recent studies have identified the choline-transporter-like proteins 1 (CTL1) and 2 (CTL2) as transporters of ethanolamine across the cellular and mitochondrial membranes (20). CTL1 and CTL2 transporters are encoded by the *SLC44A1* and *SLC44A2* genes.

### PE-plasmalogen production by the Kennedy pathway

The unique function of the CDP-Etn pathway is the formation of ether phospholipids known as plasmalogens. The synthesis of plasmalogens begins in the peroxisome, where glycerone phosphate, also known as dihydroxyacetone phosphate, is converted to alkyl-lysophosphatidic acid through a series of enzymatic steps (21–23). In the ER, alkyl-lysophosphatidic acid is converted to alkyl phosphatidic acid then to AAG (1-O-alkyl-2-acyl-sn-glycerol) (21, 24). The same way as DAG, AAG is then incorporated into the CDP-Etn Kennedy pathway (21, 25). Plasmanyl-PE is the precursor for vinyl-ether phospholipids plasmenyl-PE and plasmenyl-PC (26, 27). The Kennedy pathway is the only known producer of CDP-Etn, and the critical contributor to the total production of plasmalogens (28, 29). PE-plasmalogens are known antioxidants that are abundant in tissues with electrical activity, such as the brain, the skeletal muscle, and the heart (28, 30–35). The role of PE-plasmalogens is not completely understood, but a number of studies indicate that they are important for normal cell functions. For example, they might aid in the formation of membrane vesicles, the idea supported by their affinity for creating non-lamellar structures, their abundance in synaptic membranes and involvements in neurotransmitter homeostasis (28, 32, 36, 37). Plasmalogen deficiencies are strongly linked to neurodegeneration, and post-mortem brains of patients with Alzheimer’s disease have decreased levels of PE-plasmalogens (38–41). Additionally, PE-plasmalogens are necessary for the formation of PC-plasmalogens, which are less abundant in the cell but play an important role in cardiac tissue (42, 43). There is evidence that plasmanyl-PC could be created from AAG independently of plasmanyl-PE or CDP-Etn by choline-ethanolamine phosphotransferase *CEPT1* (44), however, current evidence indicates that plasmenyl-PE is a necessary precursor for plasmenyl-PC (21, 27). Thus, the production of CDP-Etn through the Kennedy pathway is a key regulator of plasmalogen homeostasis. Interestingly, when PE-plasmalogens are reduced, levels of PE increase so that the total amount of Etn phospholipids remains constant (45). This was demonstrated in fibroblasts from patients with Rhizomelic chondrodysplasia punctata, a peroxisomal disorder with severe plasmalogen deficiency (45). This indicates that when AAG is deficient the CDP-Etn pathway utilizes more DAG and might become upregulated when levels of plasmalogens are reduced.

### The PS decarboxylation pathway

PE synthesis in the mitochondria occurs by decarboxylation of PS (6). PS originates from PE or PC and is produced in the ER by PSS1 and PSS2 base-exchange reactions, primarily in regions that are in contact with the mitochondria, known as the mitochondria-associated membrane (MAM) (17, 46). The close physical contact between the MAM and mitochondria mediates the transport of PS into the mitochondria (1, 47, 48). Decreased ER-mitochondrial tethering decreases the PS transport into the mitochondria (49). This process is ATP-dependent, and ATP depletion in intact cells led to a decrease in PSD-regulated conversion of PS to PE (50). It is generally accepted that the transport of PS into the mitochondria is the rate-limiting step in this pathway, however, this has only been demonstrated in radiolabeling studies (51, 52). Once PS is in the mitochondria, it is converted to PE by the PSD enzyme which is encoded by the *PISD* gene. This is the only known gene that produces a PSD enzyme in mammals, and it is localized on the external side of the mitochondrial membrane (7). In yeast, however, there are two known forms of PS decarboxylases, PSD1 in the mitochondria and PSD2 in endosomes (53–55). PSD1 is the major producer of PE in the mitochondria, while PSD2 only contributes slightly to PE production in yeast cells (56). As such, it is possible that a second, less active PS decarboxylase that resides outside of the mitochondria could be identified in mammals as well.

### Relative contributions of CDP-Etn and PSD pathways

Since PE and PC are both consumed for PS synthesis and PS is consumed only for PE synthesis it remains unclear whether the CDP-Etn pathway or PSD pathway contributes more to PE production in the cell. Radiolabeling experiments in various cell lines indicate that the relative importance of each pathway might depend on the cell type, the substrate (Ser and Etn) availability, and the catalytic activity of PSD and ECT (57–59). A study in human HeLa cells showed that when the concentrations of Etn and Ser were high, 70% of PE was produced by the CDP-Eth pathway (57). At low concentrations of Etn and Ser, the CDP-Etn pathway had an even bigger role and synthesized 100-fold more PE than the PSD pathway, but at physiological concentrations of Etn and Ser, the two pathways had nearly equal contributions to PE production (57). In isolated rat hepatocytes the CDP-Etn pathway also contributed more to PE synthesis than the PSD pathway (58). On the contrary, in baby hamster kidney cells, most of PE appeared to be derived from PS in the mitochondria, and Etn did not effect PSD activity (59). These conflicting results indicate that the activity of the two PE pathways might differ depending on the organism, tissue and cell type. The variabilities in the experimental conditions should also be considered since in some of those radiolabeling experiments the lipid pools sizes were not determined (57–59). Some studies indicate that pool sizes cannot be treated as fixed parameters for metabolic flux experiments, rather they need to be measured in order to accurately estimate reaction rates (60, 61), but this idea is controversial (62). Another approach to study the importance of each pathway was possible through a targeted deletion of *PISD* and *PCYT2* genes. This was performed in various models and there is evidence that the two pathways partially compensate for one another (63–65). *Pisd* mice showed no significant difference in tissue levels of PE, PC, or PS when compared to wild-type littermates, but had a higher expression of ECT protein, suggesting that the CDP-Etn pathway was upregulated in *Pisd*^–/–^ heterozygotes (63). On the other hand, a complete hepatic deletion of *Pcyt2*, upregulated *Pisd* expression but that only partially compensated for the lack of the CDP-Etn pathway, because there was still a 50% reduction in PE levels (64). This was also the case in a muscle specific *Pcyt2* knockout mice (65). Altogether, the cell culture and animal models showed that the production of PE through the CDP-Etn pathway in the ER and PSD pathways in the mitochondria are interrelated and dependent on various extrinsic and intrinsic factors; their relative contributions to total PE is a dynamic process determined by additional conditions such as substrate levels and phospholipid transport from ER and mitochondria.

### Transport of PE between the mitochondria and ER

While a large portion of PE that is created by the PSD pathway remains in the mitochondria, particularly in the IMM, some PE is also transported out of the mitochondria to the ER (57). PE movement from the mitochondria to the ER decreases in situations when the total PE increases, indicating an ability for the transport mechanism to respond to fluctuations in PE levels (57). Therefore, it would be beneficial to establish whether the mitochondrial export of PE can be increased to compensate for *de novo* PE production in the ER, when the CDP-Etn pathway is dysfunctional. The mitochondrial PE synthesis and transport from the mitochondria to ER cannot, however, completely replace the need for the CDP-Etn pathway, as the knockout of *Pcyt2* is embryonically lethal in mice (66). Similarly, the CDP-Etn pathway is not fully capable of restoring mitochondrial PE in cases of complete PSD deficiency (63). Mutations in the PSD1 and PSD2 genes result in a yeast strain that is auxotrophic for Etn, indicating that the strain had an ability to transport PE into the mitochondria (12). However, the treatment with Etn did not restore mitochondrial PE to wild-type levels, indicating an incomplete ability for the CDP-Etn pathway to compensate for a complete deficiency in the PSD pathway (12). Similarly, mitochondrial PE in hamster ovary cells was almost entirely created by PS decarboxylation and transport of PE from the ER to the mitochondria was negligible, even when the PSD pathway was deficient (67). However, in human HeLa cells, a significant amount of PE produced by the CDP-Etn pathway was detected in the mitochondria, when ∼ 25% of mitochondrial PE was produced by the CDP-Etn pathway (57). Most investigations indicate that the CDP-Etn pathway does provide enough PE to the mitochondria to support normal function and morphology (67, 68). However, further animal and cellular studies are needed that will also take into consideration how the CDP-Eth and CDP-Cho pathways contribute to the synthesis and transport of PS from ER to the mitochondria.

### Comparison of PE molecular species made by each pathway

The nature of phospholipids is that they significantly vary structurally, based on the composition of their fatty acid sidechains. PE is no different, which is why it is beneficial to investigate differences in PE species formed by the Kennedy and PSD pathways. In a study that compared PE species in the whole cell, mitochondria, and microsomes (ER), the results showed that (16:0–18:1)PE, (18:0–18:2/18:1–18:1)PE, and (18:1–20:1)PE were 1.5–3 times more abundant in the ER than in the mitochondria (69). In the mitochondria, however, a greater percentage of PE had poly-unsaturated fatty acids (PUFAs) in the *sn*-2 position, with the most common species being (16:0–20:4)PE, (16:0–20:5)PE, (18:0–20:3)PE, (18:0–22:6)PE, and (18:1–20:5)PE. PE species made by the Kennedy pathway equilibrate between the ER and mitochondria, whereas the PE produced in the PSD pathway are mostly retained in the mitochondria. Thus, it can be assumed that the PSD pathway preferentially produces PE species with PUFAs in the *sn*-2 position, while the Kennedy pathway produces PE with mono- and di-unsaturated fatty acids in the *sn*-2 position. Considering PSS1 and PSS2 “base exchange” reactions, the only pathways to make PS later used by PSD, it is likely that PSS1 and PSS2 have preference for PC and PE species that contain PUFAs in the *sn*-2 position. This is consistent with studies with PSS2, as PSS2 showed a strong preference for PE containing docosahexaenoic acid (22:6), and some affinity for PE containing 18:1 and 20:4 in the *sn*-2 position (70). In the *sn*-1 position, PE normally contains mono-unsaturated fatty acids or saturated fatty acids regardless of which pathway it was produced by (70). A recent study in mice identified that the TLCD1/2 proteins could promote the incorporation of mono-unsaturated fatty acids at the *sn*-1 position and play a selective role in phospholipid transport between the ER and mitochondria (71).

## PE metabolism

Once created, PE has a variety of fates, some of which are summarized in Figure 1. It is mostly incorporated into cellular membranes, but it can also be remodeled and transported to other intracellular compartments or used as a source of fatty acids and DAG for the synthesis of other phospholipids (cardiolipin and phosphatidyl inositol) and glycerolipids (triglycerides, DAG, and monoglycerides) (72). Most significantly, PE is used to synthesize PC and PS, which together with PE are essential for the formation of membrane bilayers in all cells (73–77).

### PE conversion to PC

PC is the most abundant phospholipid in cell membranes (1, 78). The primary route of PC synthesis in mammals is by the CDP-Cho branch of the Kennedy pathway (78, 79). PE can also be methylated and converted into PC, but this pathway is only substantial in the liver where it makes up ∼30% of PC production (73). This reaction is catalyzed by phosphatidylethanolamine N-methyltransferase (PEMT), an enzyme that is found in the membrane of the ER and MAM and is most expressed in liver cells (74). This process helps to maintain levels of PC in hepatic membranes when dietary Cho is reduced, which is important for the maintenance of liver health, as an improper balance of PC:PE is linked to liver disease (75). Thus, PE serves an important role as a precursor for PC synthesis in the liver.

### PE conversion to PS

PS is another membrane phospholipid that is less abundant than PC or PE, as it makes up only 5–10% of the membrane phospholipids (76). However, despite its low concentration, it has important physiological functions which are due in part to its unique structure. PS has a negatively charged headgroup, which allows it to interact with different proteins than the other membrane phospholipids, and also plays a role in apoptosis (77). In mammals, PS is exclusively formed by PSS1 and PSS2 (base exchange reactions) using PC and PE phospholipids as substrates. PSS1 preferably uses PC whereas PSS2 uses PE (80–83). It is unclear whether PSS1 or PSS2 contributes more to PS production. In human HeLa cells, the knock-down of *PTDSS1* reduced PS synthesis by only 10%, while *PTDSS2* knock-down had no impact (57). In mice, both *Ptdss1* and *Ptdss2* have large roles in PS production because *Ptdss1*^–/–^ and *Ptdss2*^–/–^ mice both have significant reductions in serine exchange (84, 85). In *Ptdss2*^–/–^ mice serine exchange was decreased by 90–95%, however, the overall phospholipid content was not altered (85). Interestingly, Cho exchange was increased which indicates that *Ptdss1* activity might have increased to make up for the lack of PS being produced by the *Ptdss2* deficiency (85). However, there was no increase in *Ptdss1* mRNA expression in response to *Ptdss2* deficiency, which indicates that some other mechanism could have been responsible for maintaining the phospholipid levels, such as a reduction in PS degradation (85). Similarly, in *Ptdss1*^–/–^ mice total serine exchange activity was reduced by 85% and PS levels were unaffected in all tissues except the liver (84). It would also be important to investigate whether the conversion of PS to PE through the PSD pathway is reduced in *Ptdss1/2*^–/–^ mice, as this could potentially further explain how is PS maintained when its synthesis by *Ptdss1/2* is reduced.

## PE functions in the cell

### PE and membrane fusion/fission

Numerous studies have confirmed that PE is an essential component in membrane fusion/fission events, such as cell division (86–90). While the phospholipid is normally present on the inner membrane of cells, during the late telophase of cytokinesis, PE is more concentrated on the outer membrane at the cleavage furrow (86). Furthermore, if PE is retained on the cell surface, cytokinesis is halted, indicating that the reintegration of PE in the inner leaflet of the membrane is required to complete the process of cell division (87). This is likely due to the negative curvature that PE induces (2). When PE is in the inner membrane, the cell maintains its rounded shape, however, when PE moves to the outer membrane, the plasma membrane bends in on itself, which is a critical step for cleavage. PE has also been connected to membrane fusion (87). One study compared how different distributions of PE in the plasma membrane impacted fusion of neuroendocrine dense core vesicles and found that fusion was most efficient when PE was present in the leaflet that faced the dense core vesicle (88). When PE was absent from both sides of the membrane, the fusion pore was observed to be much less stable, which impacted the speed at which the dense core vesicles could release their contents into the cell (88). Further evidence supporting the importance of PE in membrane fusion and fission is its increased presence in synaptosomal membranes (89–92). Data was compiled from three independent studies, which demonstrated that in synaptic vesicles, PE concentration is relatively equal to PC, as PC was found to comprise 17, 32, and 25% of total lipids, while PE was shown to make up 20% of total lipids in all three studies (89–92). This indicates that PE is elevated in synaptic vesicles, because normally PC makes up 45–55% of the total phospholipids in cellular membranes while PE makes up only 15–25% (1). It should be noted that the synaptosomal PC and PE content was taken as a percentage of all lipids, not just phospholipids (89), which is why the percentages are lower. Since synaptosomes undergo frequent membrane fusion and fission, the elevated levels of PE in synaptic vesicles might indicate its contribution to those processes. To add further, our recent study demonstrated that neuronal pathways were epigenetically targeted under conditions of reduced PE synthesis by the CDP-Etn pathway (93). This was demonstrated in *Pcyt2*^+/–^ mice, which had alterations in various neuronal pathways, including the dopamine receptor signaling pathway. These changes were reversed by supplementation with phosphoethanolamine, indicating that PE plays a critical role in the dopamine signaling in neural tissues.

### PE and the mitochondrial respiration

When compared to the whole cell, mitochondria are more abundant in PE as it accounts for approximately 30% of their total membrane phospholipids (1). It is even more concentrated in the IMM, where it makes up 35–40% of total phospholipids (1). Note: these quantities were derived by averaging data from several sources, which were not explicitly listed in the review (1). However, it is generally accepted that PE has an increased concentration in the mitochondria when compared to the whole cell, which indicates that PE has an important role in mitochondrial function and morphology (94). This was demonstrated in a study that tested the impact of decreased mitochondrial PE in mammalian cells by disrupting the PSD pathway (4). The resulting PE deficient cells had decreased respiratory capacity and ATP production, as well as increased mitochondrial fragmentation (4). Additionally, studies in *Pisd* knockout mice indicated that PE is critical to mitochondrial function. *Pisd*^–/–^ mice were embryonically lethal, indicating that mitochondrial PE is a requirement for life (63). Although PE content in the mitochondria of *Pisd*^–/–^ mice was not measured, the mitochondrial PE was likely reduced, which disrupted mitochondrial morphology and respiratory function (63). Interestingly, *Pisd*^+/–^ mice had no significant decrease in mitochondrial PE when compared to wild-type mice, which helps explain why these mice were viable and had no observable differences in mitochondrial morphology or function (63).

There are numerous ways in which mitochondrial PE impacts respiration, the most obvious being its induction of negative curvature in the IMM (1, 2). This increases the surface area of the membrane, thus providing more space for the electron transport chain proteins to bind. As the number of these proteins increases, so does the mitochondria’s ability to use oxygen to create ATP (95). Additionally, decreased PE in yeast mitochondria leads to the reduced function of cytochrome c oxidase (96). Cytochrome c oxidase is one of the proton-pumping complexes, which is necessary for the development of the membrane potential that drives oxidative phosphorylation, therefore mitochondrial PE is important for cellular respiration. This is further supported by a more recent study, which demonstrated that *psd1*Δ*psd2*Δ yeast have significantly decreased function in complexes III and IV of the electron transport chain (97). Complex IV function was rescued by treatment with Etn, while complex III function was not, which indicates that in yeast, complex III relies on PE that is produced in the IMM. Finally, in mammalian cells, a decrease in mitochondrial PE disrupted the formation of complex IV (4), which facilitates the fourth step in the electron transport chain. Although cardiolipin is the primary phospholipid responsible for stabilizing these complexes in mammalian cells, there is evidence that PE has a similar role and can partially compensate for decreased cardiolipin (98–100).

Another way that mitochondrial PE might impact respiration is through its interaction with the translocase of the outer membrane (TOM) complex. The TOM complex is responsible for transporting nuclear proteins into the mitochondria, and PE deficiency has been associated with a decrease in TOM complex activity (101). In particular, PE deficient mitochondria had an impaired ability to import β -barrel proteins that reside in the outer mitochondrial membrane, such as porin (101). Studies in yeast demonstrated that a complete lack of porin resulted in a total loss of respiration (102), and more recent studies have linked porin to the import of metabolite carrier proteins into the IMM (103). Taken together, these results indicate that a lack of PE in the mitochondrial membrane might decrease the function of TOM, thus reducing the presence of porin in the OMM, which negatively impacts respiration through the decrease of mitochondrial carrier proteins. However, more studies will be needed to confirm this relationship.

### PE and the mitochondrial membrane fission and fusion

Mitochondria are dynamic organelles that form networks by fusing together and dividing. This ability is important to the metabolic health of the cell, and dysfunction in the fusion/fission machinery has been associated with neurodegenerative disease (104). Numerous studies report the impacts of PE on mitochondrial membrane morphology, with similar observations in fungi, protists, and mammals (96, 105, 106), indicating a relatively conserved role. Overall, when mitochondrial PE is reduced, it results in fragmentation of the mitochondria (fission). This has been observed in embryonic cells from *Pisd*^–/–^ mice, which had misshapen and fragmented mitochondria (63). Additionally, cells with reduced mitochondrial PE from reductions in *Pisd* or *Ptdss2* had significant levels of mitochondrial fragmentation (4). However, the specific mechanism by which PE deficiency causes fragmentation is not well-studied. One possibility is that PE influences the processing of proteins involved in membrane fusion, however, this has not been explicitly studied in mammals. We do know that long and short isoforms of human OPA1, a protein that is essential for mitochondrial fusion (107, 108) need to be co-expressed and cooperate to mediate fusion (109). In yeast, the OPA1 ortholog is Mgm1p, and its short and long isoforms must also be balanced to facilitate fusion (110). Studies have shown a connection between PE and Mgm1p processing, because *psd1*Δ yeast had decreased short isoforms and increased fragmentation of the mitochondria (111). This suggests that PE induces the production of the short isoform and thus influences mitochondrial fusion through this route (111). Regardless of the mechanism, the numerous associations between PE deficiency and mitochondrial fragmentation make it clear that PE is involved in the regulation of mitochondrial integrity (63, 96, 105, 106, 111).

### Autophagy/Mitophagy

PE is involved in autophagy, the process where cells break down damaged components and recycle the material, along with mitophagy, which is when autophagy is targeted to the mitochondria. The impact of PE on autophagy has been demonstrated in yeast, where strains with an upregulation of Psd1 had greater autophagic capacity, whereas yeast with Psd1 and Psd2 knock-outs had decreased lifespan (112). Additionally, Etn administration to mammalian cells resulted in increased PE and thus an increased autophagic flux (112). This link between PE and autophagy is likely due to its interactions with the autophagy-related protein 8 (Atg8) protein family. In particular, PE is the anchor for the microtubule-associated protein 1A/1B-light chain 3 (LC3), which is necessary for the formation of the autophagosome (113–115). PE becomes conjugated to cytosolic LC3-I to form membrane-bound LC3-II, which initiates autophagosome elongation (115, 116). Mitophagy also relies on this system, however, the autophagosome is specifically targeted to the mitochondria via receptor-dependent or ubiquitin-dependent pathways (117). One study showed that the Atg8 proteins are not required for initiating autophagosome biogenesis or targeting the mitochondria for degradation, however, they are required for proper autophagosome-lysosome fusion (118). This indicates that PE, through its interactions with the Atg8 proteins, is critical for both autophagy and mitophagy.

### Ferroptosis

Ferroptosis is the iron-dependent programmed cell death that plays a role in various diseases, such as cancer, stroke, and neurodegenerative disease (119–123). It is induced by an accumulation of reactive oxygen species which are produced through lipid peroxidation of PUFAs in cellular membranes (120). Although ferroptosis was shown to damage most phospholipid species in the cell membrane, PE species containing arachidonic acid and adrenic acid were identified as crucial regulators of ferroptosis (124). Additionally, oxidized PE, specifically 1-steaoryl-2-15-HpETE-*sn*-glycero-3-PE (SAPE-OOH), has been shown to provide the “eat-me” signal on ferroptotic cells, which interacts with the TLR2 receptor in macrophages to trigger phagocytosis (125). PE plasmalogens have also been identified as regulators of ferroptosis, as one study indicated that PUFA-containing plasmalogens are decreased in carcinoma cells, which are resistant to ferroptosis (126). These results indicate that PE and PE plasmalogens within the cell membrane play a role in the initiation of ferroptosis, as they are crucial targets for lipid peroxidation.

### PE and insulin signaling

It is thought that PE might be involved in insulin signaling, likely by modulating the fluidity of cell membranes. Insulin has been shown to influence cell membrane fluidity, which maximizes glucose transport (127, 128), and one way that the cell membrane fluidity can be increased is through decreasing PE (129). This relationship likely involves PC as well because a decrease in the ratio of PC:PE has been shown to increase insulin signaling (130). Additionally, *Drosophila* with *Pect* deficiency (the analog of *PCYT2*), develop insulin resistance and have a disrupted hunger response (131). This impact of PE on insulin signaling has also been demonstrated by our studies on *Pcyt2* knockout mice. *Pcyt2*^–/–^ mice are embryonically lethal, however, *Pcyt2*^+/–^ mice have accumulated DAG and triglycerides and demonstrate impaired glucose and insulin tolerance (132). Our subsequent studies established that the *Pcyt2*^+/–^ mouse is a model for non-alcoholic steatohepatitis (NASH) and insulin resistance, and that supplementation with phosphoethanolamine helps to reverse NASH symptoms (133). Our studies on muscle tissue in these mice indicate that *Pcyt2* deficiency significantly impacted the expression of the insulin receptor substrate 1 (IRS-1) protein, but supplementation with Cho helped restore this protein to control levels (134). It is likely that Cho supplementation helps to restore fatty acid and triglyceride homeostasis in muscle tissue, which then improves insulin signaling when PE biosynthesis is reduced (135). Altogether, these results demonstrate that PE homeostasis might influence insulin signaling in some way, either by modulating the cell membrane properties, or through its impacts on lipid metabolism.

## PE and neurological disorders

Membrane phospholipid homeostasis is important for the proper functioning of the cell. Thus, deficiencies in any of the biosynthetic pathways can be pathological (78). Over the past decade, deficiencies in the major PE-producing pathways have been linked to various diseases. In fact, a complete loss of either of the major PE-producing pathways has been shown to be embryonically lethal in mice (63, 90), which indicates that even a slight disruption in either pathway could result in negative health outcomes. This has been confirmed by the recent identification of neurological disorders that result from mutations in the genes involved in PE synthesis, including hereditary spastic paraplegia, childhood-onset neurodegeneration, and intellectual disability (8–11, 25, 135, 136–139). The influence of each disorder on PE homeostasis is summarized in Table 1.

**TABLE 1:** Summary of known neurodegenerative genetic disorders that impact PE homeostasis by downregulating a major biosynthetic pathway.

Disease name	OMIM #	Gene	Variants	Pathway	Impact on PE
Spastic paraplegia 81	618768	*SELENOI*	c.335G-C (25). c.732-2A-G (25).	CDP-Etn (Kennedy)	∼60% decrease in PE synthesis through CDP-Etn pathway (24).
Spastic paraplegia 82	618770	*PCYT2*	c.920C-T (8). P307L (8). c.730C-T (8).	CDP-Etn (Kennedy)	∼25% decrease in total cellular PE (8).
CONATOC	618868	*SLC44A1*	c.1549del (9). c.377_380del (9). 29-KB del (9).	CDP-Etn (Kennedy)	∼5% decrease in PE as% of total glycerolipids (9).
Liberfarb syndrome	618889	*PISD*	c.697 + 5G-A (10). c.830G-A (10). c.904-12_904-3delCTATCACCAC (11). c.797G-A (136).	PSD	50% decrease in PE synthesis through PSD pathway (10).
Lenz-Majewski syndrome	151050	*PTDSS1*	c.805C-T (137). c.749T-C (137). c.1058A-G (137).	Head-group base exchange	No observed difference in phospholipid levels (137).

### Hereditary spastic paraplegia in subjects with genetic variations in CDP-Etn pathway

Hereditary spastic paraplegia is a group of disorders with common features of progressive weakness and spasticity. Recent studies have identified that mutations in genes coding for enzymes in the CDP-Etn pathway can result in spastic paraplegia. The first such disease that was identified is named spastic paraplegia-81 (OMIM #618768) and is the result of a mutation in the *SELENOI* gene which codes for the EPT enzyme (135). Studies on patient fibroblasts showed decreased levels of PE species, namely PE with PUFAs were reduced, along with most plasmenyl-PE (25). Plasmanyl-PC was increased in both patient cells and *SELENOI*-KO HeLa cells, which is likely due to the decrease in conversion of AAG to plasmanyl-PE, leaving more substrate for CEPT1 to produce plasmanyl-PC (21). These studies indicate that PE ether lipid homeostasis has a significant impact on neurodevelopment.

In another recent study (8), fibroblasts from individuals with biallelic variants in *PCYT2* were analyzed. The individuals all presented with mild to severe developmental delay, epilepsy, and progressive para- or tetraparesis, named spastic paraplegia-82 (OMIM #618770). Lipid analysis showed decreased levels of PC, PE, lyso-PE, and the PE plasmalogens, while levels of PC plasmalogens were increased. Additionally, since the Kennedy pathway uses DAG, this molecule and its ether lipid accumulated, which resulted in increased synthesis of triglyceride ether lipids. This indicates that plasmalogen metabolism is significantly affected by *PCYT2* deficiencies, which is not surprising considering that CDP-Etn is required for the formation of PE plasmalogens. There was some evidence that the PSD pathway can also synthesize PE plasmalogens when the CDP-Etn Kennedy pathway is deficient, by the *PTDSS1* mediated conversion of plasmenyl-PC to plasmenyl-PS, followed by decarboxylation to form plasmenyl-PE. However, since most PC and PS ether lipids can only be produced from PE ether lipids, with the exception of plasmanyl-PC (25, 28), patients with biallelic *PCYT2* variants must have some ECT activity to account for the presence of plasmalogens (8). Overall, these secondary changes in phospholipid metabolism resulted in a progressive gray-matter disorder, with little to no functional issues in other tissues, showing the importance of PE for neurons and the demand for ether phospholipids in the brain. Therefore, the imbalance of membrane lipids is likely what causes the neurodegeneration, either by reducing neuronal function, or by the neurotoxicity of lipid accumulation.

In patients with either *SELENOI* or *PCYT2* variants, fibroblasts analysis showed decreased levels of PUFA-containing PE (8, 25), which is surprising considering that the PSD pathway produces PE with more PUFA species in the sn-2 position (69). Further investigation into how functional the *SELENOI* and *PCYT2* variants are in these patients would be helpful in explaining this result and would elucidate the extent to which the PSD pathway compensates for the lack of PE being produced through the CDP-Etn Kennedy pathway. The researchers studying *PCYT2* deficiency suggested that treatment with Cho or Ser could supplement alternative pathways (i.e., CDP-Cho production of PC, base exchange of PC to form PS, and subsequent production of PE through PSD) and help restore the membrane phospholipid balance (8). It would be necessary to study Cho and Ser supplementation to gain insight into whether an increased availability of either nutrient would impact phospholipid levels. Even if Ser and Cho supplementation can improve phospholipid balance, it is unlikely that it would restore ether lipids since plasmenyl-PC and -PS can only be produced from PE ether lipids; PE ether lipids are only produced *de novo* by the CDP-Etn Kennedy pathway, which is impaired in the patients. Therefore, a supplementation with PE ether lipids is a must in patients with *PCYT2* and *SELENOI* mutant variants.

### Childhood-onset neurodegenerative disease in patients with SLC44A1 variants

Our recent study identified a novel childhood-onset neurodegenerative disease named by OMIM as CONATOC (OMIM #618868) that is caused by Choline-transporter like protein 1 (CTL1) deficiency (9). Four individuals with neurogenerative disease were found to have homozygous mutations in the *SLC44A1* gene encoding CTL1. The *SLC44A1* variants identified are c.1549del, p.Asp517Metfs*19, c.377_380del, p.Ser126Metfs*8, and a 29 kb deletion (including exome 3) resulting in Lys90Metfs*18.

The patients presented with numerous clinical features, including cognitive decline, dysphagia, optic atrophy, progressive ataxia, dysarthria, and incontinence. Since the transport of exogenous Cho is necessary for PC production through the CDP-Cho branch of the Kennedy pathway, it was expected that PC would be diminished in patient fibroblasts, however, it was preserved. The results indicated that PC synthesis could be partially compensated by upregulating alternate biosynthetic pathways. For example, since PC can be created by the methylation of PE, the enzyme that catalyzes this reaction (PEMT) was expressed 25% more in mutant cells. Thus, it is not surprising that this study also found that mutant cells were significantly deficient in PE and PS, and that the CDP-Etn branch of the Kennedy pathway was reduced. The downregulation of the CDP-Etn pathway can be explained by our subsequent study, which showed that CTL1 is also a transporter of Etn (20). We demonstrated that there is a direct link between CTL1 expression and PE synthesis, which further explains the pathology of CTL1 deficiencies. Interestingly, the CTL1 deficient fibroblasts from individuals with CONATOC are able to transport Etn and Cho through an alternate transporter, CTL2 (20). CTL2 was shown to have a decreased affinity for Etn and Cho when compared to CTL1, however, Cho supplementation rescued phospholipids (including PE and PS) and restored mitochondrial morphology. This discovery implies that future work should be done to test whether supplementation with Etn would also help to increase phospholipid levels in these patients.

### Intellectual disability in patients with PISD variants

Another neurological disorder, named Liberfarb syndrome (OMIM #618889), has recently been linked to PE as it results from mutations in the *PISD* gene (10). Two patients with white matter changes, along with other clinical features, were found to have heterozygous variants in the *PISD* gene—a splicing variant in intron 5 c.697 + 5G > A, p.(?) and a missense variant in exon 8 c.830G > A, p.Arg277Gln. Fibroblasts from these patients had a 50% decrease in synthesis of PE from the PSD pathway, which modified the mitochondrial respiration and morphology. Another study looked at four individuals who shared many neurological features, including microcephaly, intellectual disability, retinal degradation, and sensorineural hearing loss, and found a common deletion mutation of 10 base pairs from the −3 to −12 position in intron 8 of the *PISD* gene c.904-12_904-3delCTATCACCAC p.(?) (11). All four individuals were homozygous for this rare variant, which is a result of non-random mating. Although the individuals were homozygous for the mutation, the researchers demonstrated that some *PISD* activity was retained as the variant gene still produces some full-length transcripts, which explains why this genotype is not lethal. Unfortunately, this study did not investigate phospholipid concentrations in these patients but given the results of other studies with reduced PSD pathways, mitochondrial PE might be depleted (10, 12, 138).

### Intellectual disability in patients with PTDSS1 variants

Lenz-Majewski syndrome (OMIM #151050) is characterized by a “gain-of-function” mutation in the *PTDSS1* gene which produces PS from PC (137). Although this gene is not directly involved in the production of PE, it produces PS which can be used in the PSD pathway. Clinical features of this disease include intellectual disability, osteosclerosis, and craniofacial and distal-limb abnormalities (137). Various missense variants have been identified including c.805C > T resulting in p.Pro269Ser, c.749T > C resulting in p.Leu265Pro, and c.1058A > G resulting in p.Gln353Arg (137). Studies on patient fibroblasts demonstrated that these missense mutations caused a reduction in the enzyme end-product (PS) inhibition (137). Interestingly, there were no significant differences in phospholipid content between the patient and control fibroblasts (137). Considering that *PTDSS1* has a direct impact on phospholipid homeostasis the compensatory mechanisms are to be determined in those patients, including lipid alterations in various tissues and specific organelles.

## Methods of modifying PE levels

The connection between PE and neurological function is well established, as seen through the neurological disorders that result from dysfunctions in the genes involved in PE biosynthesis. As such, it is beneficial to investigate methods of increasing PE levels in individuals who are affected by these devastating disorders. Unfortunately, there is no known cure for the above-mentioned disorders, however, there have been some investigations into the potential mechanisms for increasing PE.

### Compensation from the other major PE biosynthetic pathway

One of the ways of increasing PE production is the upregulation of the alternative biosynthetic pathway not affected by a disease gene. The ability of the Kennedy and PSD pathways to somewhat compensate for one another is documented and discussed in the section about the relative contributions of the pathways (57, 63), however, it is apparent that neither pathway could be fully compensatory. This is demonstrated by the studies done in mice, which showed that complete knockout of either pathway was lethal (63, 66). However, in studies using mice that are heterozygous for mutations in either *Pisd* or *Pcyt2*, it was observed that the alternate pathway can help supplement the deficiencies in the other. For example, in *Pisd*^+/–^ mice, ECT activity was increased by 35–40% (63). In contrast, *Pcyt2*^+/–^ mice were not shown to have any increased activity in the PSD pathway, yet they maintained regular PE levels due to decreased degradation (66). Although the PSD pathway might not be upregulated during *PCYT2* deficiency, there is evidence that mitochondrial PE can be transported into the ER when cellular PE is decreased (57). Additionally, the PSD pathway can synthesize PE plasmalogens when ECT is deficient (8). Together, these results indicate that a targeted upregulation of either pathway might help restore PE to some degree.

### Supplementing ethanolamine to upregulate the Kennedy pathway

Etn is a part of the human diet, and it is rich in foods such as lemon grass, daikon radish, caraway, and muscadine grape, according to the FooDB database (140). Since the CDP-Etn pathway relies on exogenous Etn to produce PE, supplementing with Etn might help increase the activity through this pathway when the PSD pathway is deficient. This has been tested in both yeast and humans, and the results are summarized in Table 2. Early studies of Etn supplementation were done on *psd1*Δ yeast, which had decreased mitochondrial respiration and increased ER stress (141). When this strain was supplemented with Etn, production of PE was increased through the Kennedy pathway, which helped reduce ER stress but did not have an impact on respiration. This indicates that the restorative ability of Etn supplementation has limitations, which might be related to the different species of PE produced in the Kennedy and PSD pathways (69). It is possible that PE with PUFAs in the *sn*-2 position are required for the proper functioning of the mitochondria, but this has not been investigated directly. Another study looked at whether Etn supplementation could help rescue cells from humans with *PISD* mutations (136). The individuals in this study had spondylo-epi-metaphyseal dysplasia’s (SEMD), and analysis of their fibroblasts showed fragmentation of the mitochondria. Supplementing patient cells with Etn alone did not produce any noticeable differences in cellular viability. However, when the cells were also treated with MG-132 to induce a stress response, Etn treatments helped increase cell viability, likely by increasing mitochondrial PE and thus reducing apoptosis. There is evidence that mitochondrial fragmentation leads to an overproduction of reactive oxygen species, which can cause cell death (142, 143). Therefore, the fact that Etn supplementation reduced apoptosis when a stress response was triggered suggests that the Etn supplementation may have improved the cells viability by increasing mitochondrial PE, which is associated with mitochondrial integrity (63, 96, 105, 107, 111). However, since PE content in the cell and mitochondria was not directly measured, further study is needed to conclude that Etn supplementation increases PE production through the Kennedy pathway in cells that are deficient in *PISD*. Etn supplementation might also help to increase PE synthesis in patients with childhood-onset neurodegenerative disease due to mutations in the high-affinity Etn transporter, *SLC44A1*/CTL1 (9). This is indicated in our recent study (20), which found that the second transporter CTL2 may supply Etn. CTL2 has a low affinity and requires a higher concentration of Etn to effectively transport it into the cell. Thus, if Etn concentration is raised via supplementation, then Etn transported by CTL2 might be sufficient to elevate PE synthesis through the CDP-Etn Kennedy pathway. This would need to be assessed further by treating CTL1 deficient fibroblasts with Etn to see whether it has any impact on PE levels. It is important to note that the impact of Etn supplementation on PE homeostasis has not been studied *in vivo* in animals or humans, so even though Etn seems to improve the viability of *PISD* deficient cells, we are a long way from understanding if and how this could be accomplished in patients.

**TABLE 2:** Summary of studies that investigated the impact of Etn and lyso-Etn supplementation on PL homeostasis.

Supplement	References	Organism	Genetic variant	Treatment	Impact
Etn	Riekhof and Voelker (12).	Yeast	*psd1*Δ*psd2*Δ	5 mM	Normal growth, shows that Kennedy pathway can supply sufficient PE when supplemented with Etn.
			*psd1*Δ*psd2*Δ*ect1*Δ	5 mM	Insufficient PL biosynthesis due to inactive Kennedy pathway, strain not viable.
	Girisha et al. (136).	Human Fibroblasts	Homozygous *PISD* missense variant: [p.Cys266Tyr]	1 mM	No increase in viability.
				1 mM + 20μM MG-132	Increased viability compared to treatment with MG-132 only, indicates ability for Etn supplementation to reduce activity of apoptotic proteases.
Lyso-PE	Riekhof and Voelker (12).	Yeast	*psd1*Δ*psd2*Δ	0.5 mM	Normal growth, mitochondrial pool of PE replenished better than treatment with Etn.
			*psd1*Δ*psd2*Δ*ect1*Δ	0.5 mM	Normal growth, shows that lyso-PE use is independent of the Kennedy pathway.
	Tasseva et al. (4).	Chinese Hamster Ovary	Deficiency in *Ptdss2* resulting in 95% decrease in PS synthesis and chronically reduced mitochondrial PE synthesis	0.1 mM	Improvement in cell growth, mitochondrial morphology, and ATP production.
	Zhao et al. (10).	Human Fibroblasts	*PISD* splicing variant in intron 5: [p.(?)] *PISD* missense variant in exon 8: [p.Arg277Gln]	0.05 mM	Mitochondrial and lysosomal morphology restored.

### Induction of the mitochondrial PSD pathway

Another option for upregulating PE production is through an induction of the PSD pathway. This was investigated in *Drosophila*, and the researchers found that when the Kennedy pathway is disrupted, induction of the PSD pathway restored cellular PE levels (144). *Drosophila* with mutations in the *Pect* gene, which is equivalent to *PCYT2* in humans, had abnormal phospholipid composition, retinal degradation, and defective visual responses. When the PSD pathway was induced in these mutants, they no longer displayed abnormalities in their vision system and cellular PE was restored. The researchers also demonstrated that contact between the ER and mitochondria (through ER MAM) is necessary to transport the PE out of the mitochondria for use in the whole cell. Overall, this demonstrates some potential for inducing *PISD* genes in humans to treat deficiencies in *PCYT2*, so long as PE transport from the mitochondria to ER is undisrupted and supported by functional MAM’s.

### Stimulation of lyso-PE acylation

Although the lyso-PE acylation pathway has a minor contribution to cellular PE when compared to the CDP-Etn and PSD pathways, there is some evidence that supplementing with small amounts of lyso-PE can increase PE levels. Table 2 summarizes the studies that have demonstrated this in both yeast and human cells (10, 12). The yeast strains with PSD1 and PSD2 mutations were auxotrophic for Etn, which indicated a reliance on the Kennedy pathway for PE production (12). PE produced by the Kennedy pathway could not restore mitochondrial lipid composition (68), indicating a need to identify a pathway that better supports cellular PE. The treatment of PSD mutant yeast with exogenous lyso-PE supplied PE to mitochondria (12). Lyso-PE was much more effective than Etn when the yeast was grown under respiratory conditions, where mitochondrial number and size naturally increase. This ability for lyso-PE to better support growth under respiratory conditions indicates that PE produced by lyso-PE acylation supports mitochondrial function better than PE made by the Kennedy pathway.

This has also been demonstrated in mammalian cells. The lyso-PE treatment improved cell growth, mitochondrial morphology, and ATP production in *Ptdss2* deficient hamster ovary cells (4). These cells had a significantly decreased PS synthesis (145) and reduced mitochondrial PE (4). Lyso-PE treatment of *PISD* mutant human fibroblasts reduced mitochondrial fragmentation and rescued the cell from apoptosis (10). Although these studies make lyso-PE appear promising treatment option, there are some limitations to consider. The lyso-PE acylation pathway cannot restore PE plasmalogens, since they are exclusively produced by the CDP-Etn pathway (28, 29) and it might not be able to rescue the phenotypes caused by defects in the Kennedy pathway. It is also important to consider whether supplementing lyso-PE is safe because some evidence indicates that it might stimulate lipid accumulation in the liver (146) and because of its detergent properties, it could damage cellular membranes (147). In livestock animals dietary lyso-phospholipids improved nutrient absorption, but these experiments did not measure lipid levels or toxicity (148–150). Overall, there is a lack of clinical evidence to suggest that lyso-PE supplementation would be a method of choice for restoring PEs in human deficiencies caused by mutations in *PISD* and the genes from the CDP-Etn Kennedy pathway.

There are several proteins that are involved in the uptake and utilization of lyso-PE in yeast. Lyso-PE is transported by Dnf1p and Dnf2p, which are ATP-dependent amino phospholipid translocases (12), and Ale1p is the enzyme that catalyzes the acylation of yeast lyso-PE (151). These findings set the groundwork for investigations into whether there are similar pathways in humans that could be manipulated to help increase PE in deficient patients. In humans, plasma lyso-PE is much higher than in cells and tissues (152), suggesting that an upregulation of lyso-PE transport might help to increase lyso-PE acylation within the cell. The mechanisms for lyso-PE transport are not well-understood, however, the Mfsd2a transporter, which primarily transports lyso-PC, is known to also interact with lyso-PE (14). This protein, along with other potential transporters, need to be further investigated in order to determine how the cellular import of lyso-PE could be stimulated. Once inside the cell, lyso-PE is converted to PE by lysophospholipid acyltransferases. The specific acyltransferases that are involved in PE synthesis have been identified as LPEAT1, LPCAT4, and LPCAT3 (13). Therefore, an increase in any of these activities would potentially increase PE production from lyso-PE. Altogether, a great deal of research is needed to determine whether a targeted upregulation of any part of the lyso-PE pathway is feasible and safe in humans.

### Supplementation of PC, PE, and PS

The impact of supplementation of PC, PE, and PS on phospholipid homeostasis is not well-studied, however, it is expected that dietary phospholipids will modify fatty acid composition and balance membrane lipid ratio. In many studies where phospholipid supplementation was studied, there is no documented analysis of the membrane phospholipid content or composition, rather the focus is on other physiological impacts (153–157). For example, in a study that investigated dietary supplementation of PC and vitamin B_12_ in mice, there was no observed impact on phospholipid subclasses, however, supplementation improved learning behavior by increasing docohexaenoic (DHA) acid content in the brain (153). Similarly, dietary PS supplementation in humans improved cognitive function, however, the phospholipid compositions of the test subjects was not studied. Dietary PS increased circulating PS levels but how the supplementation impacted other phospholipids is not known (154–158). A recent study in a SAMP8 mice (a model for cognitive deficiency) looked at how supplementing with DHA-containing PC and PS could restore lipid homeostasis (159). This study analyzed the lipid content of the cerebral cortex and found that DHA-PS and DHA-PC did not alter major phospholipids, however, increased the levels of lyso-PE. After dietary intake, PE is hydrolyzed to lyso-PE or Etn before absorption into the intestinal mucosa (160). Once absorbed, Etn is transported to the portal vein where it can be used to produce PE through the CDP-Etn pathway (160). Intestinal lyso-PE is typically re acylated to PE, but it could also be further degraded to form Etn, which could then enter the CDP-Etn pathway (12). Altogether, these studies indicate that dietary PE might increase lyso-PE and Etn which can be used to reform PE, but this has not been investigated directly. Intervention studies would be needed to confirm whether dietary phospholipids help to balance cellular PE levels.

### Supplementation of plasmalogens

The idea of supplementing plasmalogens has been studied as a treatment method for Alzheimer’s patients, who have significantly reduced levels of plasmalogens (38–41). Since plasmalogens are also reduced in patients with dysfunctional CDP-Etn pathways, namely spastic paraplegia 81 and 82, plasmalogen supplementation might also be of benefit to them. Oral supplementation of plasmalogens and their derivatives has been shown to improve cognitive function in Alzheimer’s disease rodent models (161, 162). Some benefits of plasmalogen supplementation have also been observed in a clinical study on human Alzheimer’s patients (163). Interestingly, for the human trial, plasmalogen levels did not increase after supplementation, even though some improvements in cognitive function were noted (163). It is possible that plasmalogen levels did increase enough to have a biological role but the difference was not significant enough to be measurable (21, 164). Something to consider when supplementing with plasmalogens is that they might undergo structural changes during digestion. This was suggested by a study in rats, where PE-plasmalogens underwent structural changes in the intestinal mucosa (165). Therefore, more studies are needed to determine whether supplementation would help increase plasmalogen levels in patients with deficiencies in the CDP-Etn pathway.

### Reducing PEMT conversion of PE to PC

A mechanism that could potentially increase tissue PE levels is to decrease the PEMT mediated liver conversion of PE to PC, which has been accomplished by drugs that induce peroxisome proliferation or inhibit cellular methylation (166–169). Peroxisome proliferators are drugs that are known to induce the proliferation of peroxisomes, mitochondria, and ER in rodent hepatocytes (170–173). Clofibric acid, tiadenol, and di-(2-ethylhexyl) phthalate are peroxisome proliferators (fibrates) that could increase PE and PC levels in the liver (165–167). Following fibrate treatments, CTP:phosphocholine cytidylyltransferase and PSD activity were increased, while PEMT and CTP:phosphoethanolamine cytidylyltransferase (ECT/*Pcyt2*) activity were suppressed (163). This indicates that fibrates might increase PE levels by increasing PSD pathway and by reducing PE methylation to PC (168). This does not result in decreased PC levels because PC synthesis is increased through the CDP-Cho pathway, as indicated by increased activity of the rate-limiting enzyme, CTP:phosphocholine cytidylyltransferase (168). This is an unexplored approach for increasing liver PE and PC and future studies are needed to probe whether fibrates could be beneficial for repairing membrane phospholipids in the other tissues.

Another drug that has been studied is a methylation inhibitor called 3-deazaadenosine (169). Administration of this drug to rats intravenously and to rat hepatocytes in cell culture resulted in decreased conversion of PE to PC, as well as an increase in conversion of phosphocholine to CDP-choline (which is mediated by the CTP: phosphocholine cytidylyltransferase) (169). This showed that the PEMT and CDP-Cho Kennedy pathways are coordinately regulated, such that when PEMT activity is decreased, PC production through the Kennedy pathway increases. This has also been demonstrated in PEMT knock-out mice, which had a 60% increase in CTP:phosphocholine cytidylyltransferase activity (174). Additional studies on the PEMT deficient mice showed that they had no significant differences in development, behavior, or fecundity when compared to their wildtype littermates (175), however, they develop a severe hepatic steatosis under choline deficiency but also when supplemented with dietary choline (174–176). Therefore, before targeting PEMT pathway to preserve PE levels without decreasing PC content in the liver it is important to consider whether this would contribute to fatty liver development.

Peroxisome proliferators have been studied as a treatment method for Alzheimer’s disease, and although they do not appear to cross the blood-brain barrier, they still improve cognitive function through some unknown mechanism (177, 178). PEMT is much less abundant in the brain than it is in the liver. In rats, the brain has approximately 1% of the amount of PEMT found in the liver (179). However, PEMT activity could be increased in the brain tissue of rats with choline deficiency (180). Other studies demonstrated that PEMT has a role in brain development (181, 182) showing that PEMT-deficient mice had significantly decreased fetal brain PC (182). However, the neonatal development and conditions of choline deficiency were not relevant to the PE-related genetic disorders discussed here. It is generally accepted that PEMT is much less active in the brain than in the liver (178, 183). According to the Human Protein (Brain) Atlas, PEMT mRNA is universally distributed in the human brain but the expression is about 20-fold lower than in the liver (184). Therefore, it is highly questionable whether a reduction of PEMT activity would have any effect on PE homeostasis in neural tissues.

A possible benefit of downregulating PEMT is the observed increase in PC production through the Kennedy pathway (168, 169, 174). This pathway could replenish some plasmalogens by using CDP-Cho as a substrate, rather than CDP-Etn (44). However, this is somewhat speculative as plasmalogen production was not studied in animal models, so future studies are needed to confirm whether this would occur. Even if plasmalogen production is increased in the liver, further studies would be needed to determine whether this has any impact on plasmalogen levels in other tissues.

## Conclusion

The cellular regulation of PE homeostasis is a complex process, and its recent connection to neurodegenerative diseases provides motivation to better understand the many systems involved. Although recent studies point to potential treatment options for patients with dysfunctions in PE synthesis, most studies have focused on pinpointing the disease genes or elucidating the elements involved in PE regulation. This is a promising first step; however, future studies must be conducted to investigate the effectiveness and feasibility of the treatment options that were proposed in this review. Of course, there could not be one agreed upon method of increasing specific PE species for all individuals who are deficient. This is made clear by the various disease genes that lead to decreased PE levels, and the differences in their pathologies. However, the ability of lyso-PE acylation to supply both the cellular and mitochondrial pools of PE make it an interesting target for future studies, especially since there have yet to be any disease genes associated with this pathway. As such, it has the potential to restore PE homeostasis in patients with dysfunction in either the Kennedy or PSD pathways, but our current knowledge of how to increase lyso-PE acylation *in vivo* is extremely limited. A more realistic approach would be to assess the impacts of dietary phospholipid supplements, along with drugs that are known to impact phospholipid homeostasis, in order to find therapeutic methods that could help the individuals who are currently suffering.

## Author contributions

MS: writing—original draft. MS, RI, and MB: writing—review and editing. All authors approved the final manuscript.

## References

[1] Vance JE. Phospholipid synthesis and transport in mammalian cells. *Traffic.* (2015) 16:1–18. 10.1111/tra.12230 25243850

[2] Lessen HJ Sapp KC Beaven AH Ashkar R Sodt AJ. Molecular mechanisms of spontaneous curvature and softening in complex lipid bilayer mixtures. *Biophys J.* (2022) 121:3188–99. 10.1101/2022.02.17.480963 35927953 PMC9463698

[3] Dowhan W Bogdanov M. Lipid-dependent membrane protein topogenesis. *Ann Rev Biochem.* (2009) 78:515–40. 10.1146/annurev.biochem.77.060806.091251 19489728 PMC3033430

[4] Tasseva G Bai HD Davidescu M Haromy A Michelakis E Vance JE. Phosphatidylethanolamine deficiency in mammalian mitochondria impairs oxidative phosphorylation and alters mitochondrial morphology. *J Biol Chem.* (2013) 288:4158–73. 10.1074/jbc.m112.434183 23250747 PMC3567666

[5] Kennedy EP Weiss SB. The function of cytidine coenzymes in the biosynthesis of phospholipides. *J Biol Chem.* (1956) 222:193–214. 10.1016/s0021-925850785-2 13366993

[6] Borkenhagen LF Kennedy EP Fielding L. Enzymatic formation and decarboxylation of phosphatidylserine. *J Biol Chem.* (1961) 236:63319. 10.1016/s0021-925863319-3

[7] Zborowski J Dygas A Wojtczak L. Phosphatidylserine decarboxylase is located on the external side of the inner mitochondrial membrane. *FEBS Lett.* (1983) 157:179–82. 10.1016/0014-579381141-7 6862014

[8] Vaz FM McDermott JH Alders M Wortmann SB Kölker S Pras-Raves ML Mutations in PCYT2 disrupt etherlipid biosynthesis and cause a complex hereditary spastic paraplegia. *Brain.* (2019) 142:3382–97. 10.1093/brain/awz291 31637422 PMC6821184

[9] Fagerberg CR Taylor A Distelmaier F Schrøder HD Kibæk M Wieczorek D Choline transporter-like 1 deficiency causes a new type of childhood-onset neurodegeneration. *Brain.* (2019) 143:94–111. 10.1093/brain/awz376 31855247

[10] Zhao T Goedhart CM Sam PN Sabouny R Lingrell S Cornish AJ PISD is a mitochondrial disease gene causing skeletal dysplasia, cataracts, and white matter changes. *Life Sci Allian.* (2019) 2:353. 10.26508/lsa.201900353 30858161 PMC6412922

[11] Peter VG Quinodoz M Pinto-Basto J Sousa SB Di Gioia SA Soares G The liberfarb syndrome, a multisystem disorder affecting eye, ear, bone, and brain development, is caused by a founder pathogenic variant in the PISD gene. *Genet Med.* (2019) 21:2734–43. 10.1038/s41436-019-0595-x 31263216 PMC6892740

[12] Riekhof WR Voelker DR. Uptake and utilization of lyso-phosphatidylethanolamine by saccharomyces cerevisiae. *J Biol Chem.* (2006) 281:36588–96. 10.1074/jbc.m608851200 17015438

[13] Hishikawa D Shindou H Kobayashi S Nakanishi H Taguchi R Shimizu T. Discovery of a lysophospholipid acyltransferase family essential for membrane asymmetry and diversity. *Proc Natl Acad Sci USA.* (2008) 105:2830–5. 10.1073/pnas.0712245105 18287005 PMC2268545

[14] Nguyen LN Ma D Shui G Wong P Cazenave-Gassiot A Zhang X Mfsd2a is a transporter for the essential omega-3 fatty acid docosahexaenoic acid. *Nature.* (2014) 509:503–6. 10.1038/nature13241 24828044

[15] Schober C Schiller J Pinker F Hengstler JG Fuchs B. Lysophosphatidylethanolamine is – in contrast to – choline – generated under in vivo conditions exclusively by phospholipase A2 but not by hypochlorous acid. *Bioorganic Chem.* (2009) 37:202–10. 10.1016/j.bioorg.2009.09.002 19818468

[16] Sundler R Åkesson B Nilsson Å. Quantitative role of base exchange in phosphatidylethanolamine synthesis in isolated rat hepatocytes. *FEBS Lett.* (1974) 43:303–7. 10.1016/0014-579380667-8 4213338

[17] Stone S Vance JE. Phosphatidylserine synthase-1 and -2 are localized to mitochondria-associated membranes. *J Biol Chem.* (2000) 275:34534–40. 10.1074/jbc.M002865200 10938271

[18] Sundler R Akesson B. Regulation of phospholipid biosynthesis in isolated rat hepatocytes. Effect of different substrates. *J Biol Chem.* (1975) 250:3359–67. 10.1016/s0021-925841523-8 1123345

[19] Gibellini F Smith TK. The Kennedy pathway-de novo synthesis of phosphatidylethanolamine and phosphatidylcholine. *IUBMB Life.* (2010) 62:354. 10.1002/iub.354 20503434

[20] Taylor A Grapentine S Ichhpuniani J Bakovic M. Choline transporter-like proteins 1 and 2 are newly identified plasma membrane and mitochondrial ethanolamine transporters. *J Biol Chem.* (2021) 296:100604. 10.1016/j.jbc.2021.100604 33789160 PMC8081925

[21] Dorninger F Werner ER Berger J Watschinger K. Regulation of plasmalogen metabolism and traffic in mammals: the fog begins to lift. *Front Cell Dev Biol.* (2022) 10:946393. 10.3389/fcell.2022.946393 36120579 PMC9471318

[22] Thai T-P Heid H Rackwitz H-R Hunziker A Gorgas K Just WW. Ether lipid biosynthesis: isolation and molecular characterization of human dihydroxyacetonephosphate acyltransferase. *FEBS Lett.* (1997) 420:205–11. 10.1016/s0014-579301495-6 9459311

[23] Ofman R. Acyl-COA:dihydroxyacetonephosphate acyltransferase: cloning of the human cdna and resolution of the molecular basis in rhizomelic chondrodysplasia punctata type 2. *Hum Mol Genet.* (1998) 7:847–53. 10.1093/hmg/7.5.847 9536089

[24] Lodhi IJ Yin L Jensen-Urstad APL Funai K Coleman T Baird JH Inhibiting adipose tissue lipogenesis reprograms thermogenesis and PPARΓ activation to decrease diet-induced obesity. *Cell Metab.* (2012) 16:189–201. 10.1016/j.cmet.2012.06.013 22863804 PMC3467338

[25] Horibata Y Elpeleg O Eran A Hirabayashi Y Savitzki D Tal G Ept1 (selenoprotein I) is critical for the neural development and maintenance of plasmalogen in humans. *J Lipid Res.* (2018) 59:1015–26. 10.1194/jlr.p081620 29500230 PMC5983406

[26] Gallego-García A Monera-Girona AJ Pajares-Martínez E Bastida-Martínez E Pérez-Castaño R Iniesta AA A bacterial light response reveals an orphan desaturase for human plasmalogen synthesis. *Science.* (2019) 366:128–32. 10.1126/science.aay1436 31604315

[27] Lee T-C Qian C Snyder F. Biosynthesis of choline plasmalogens in neonatal rat myocytes. *Arch Biochem Biophys.* (1991) 286:498–503. 10.1016/0003-986190071-p 1897971

[28] Bakovic M Fullerton MD Michel V. Metabolic and molecular aspects of ethanolamine phospholipid biosynthesis: the role of CTP:phosphoethanolamine cytidylyltransferase (PCYT2). *Biochem Cell Biol.* (2007) 85:283–300. 10.1139/o07-006 17612623

[29] Arthur G Page L. Synthesis of phosphatidylethanolamine and ethanolamine plasmalogen by the CDP-ethanolamine and decarboxylase pathways in rat heart, kidney and liver. *Biochem J.* (1991) 273:121–5. 10.1042/bj2730121 1989575 PMC1149888

[30] Balakrishnan S Goodwin H Cumings JN. The distribution of phosphorus-containing lipid compounds in the human brain. *J Neurochem.* (1961) 8:276–84. 10.1111/j.1471-4159.1961.tb13553.x 13864239

[31] DeVries GH Zetusky WJ Zmachinski C Calabrese VP. Lipid composition of axolemma-enriched fractions from human brains. *J Lipid Res.* (1981) 22:208–16. 10.1016/s0022-227535364-5 7240954

[32] Gremo F De Medio GE Trovarelli G Dessi S Porru S. Mature and immature synaptosomal membranes have a different lipid composition. *Neurochem Res.* (1985) 10:133–44. 10.1007/bf00964778 3982588

[33] Okano G Matsuzaka H Shimojo T. A comparative study of the lipid composition of white, intermediate, red and heart muscle in rats. *Biochim Et Biophys Acta (BBA) Lipids Lipid Metab.* (1980) 619:167–75. 10.1016/0005-276090252-0 7417465

[34] Waku K Uda Y Nakazawa Y. Lipid composition in rabbit sarcoplasmic reticulum and occurrence of alkyl ether phospholipids. *J Biochem.* (1971) 69:483–91. 10.1093/oxfordjournals.jbchem.a129491 4323968

[35] Yamada K Imura K Taniguchi M Sakagami T. Studies on the composition of phospholipids in rat small intestinal smooth muscle1. *J Biochem.* (1976) 79:809–17. 10.1093/oxfordjournals.jbchem.a131134 931979

[36] West A Klauda JB Sachs JN Tristam-Nagle S Gawrisch K Vanni S How do ethanolamine plasmalogens contribute to order and structure of neurological membranes? *Biol Chem.* (2020) 2020:8850. 10.1021/acs.jpcb.9b08850.s001 PMC8157475 31916765

[37] Dorninger F König T Scholze P Berger ML Zeitler G Wiesinger C Disturbed neurotransmitter homeostasis in ether lipid deficiency. *Hum Mol Genet.* (2019) 28:2046–61. 10.1093/hmg/ddz040 30759250 PMC6548223

[38] Wood PL Barnette BL Kaye JA Quinn JF Woltjer RL. Non-targeted lipidomics of CSF and frontal cortex grey and white matter in control, mild cognitive impairment, and alzheimer’s disease subjects. *Acta Neuropsychiatr.* (2015) 27:270–8. 10.1017/neu.2015.18 25858158

[39] Han X Holtzman DM McKeel DW. Plasmalogen deficiency in early alzheimer’s disease subjects and in animal models: molecular characterization using electrospray ionization mass spectrometry. *J Neurochem.* (2001) 77:1168–80. 10.1046/j.1471-4159.2001.00332.x 11359882

[40] Ellison DW Beal MF Martin JB. Phosphoethanolamine and ethanolamine are decreased in alzheimer’s disease and huntington’s disease. *Brain Res.* (1987) 417:389–92. 10.1016/0006-899390471-9 2958109

[41] Rothhaar TL Grösgen S Haupenthal VJ Burg VK Hundsdörfer B Mett J Plasmalogens inhibit APP processing by directly affecting γ-secretase activity in alzheimer’s disease. *Sci World J.* (2012) 2012:1–15. 10.1100/2012/141240 22547976 PMC3322458

[42] Lee TC. Biosynthesis and possible biological functions of plasmalogens. *Biochim Et Biophys Acta (BBA) Lipids Lipid Metab.* (1998) 1394:129–45. 10.1016/s0005-276000107-6 9795186

[43] Tham YK Huynh K Mellett NA Henstridge DC Kiriazis H Ooi JYY Distinct lipidomic profiles in models of physiological and pathological cardiac remodeling, and potential therapeutic strategies. *Biochim Et Biophys Acta (BBA) Mol Cell Biol Lip.* (2018) 1863:219–34. 10.1016/j.bbalip.2017.12.003 29217479

[44] Horibata Y Sugimoto H. Differential contributions of choline phosphotransferases CPT1 and CEPT1 to the biosynthesis of choline phospholipids. *J Lip Res.* (2021) 62:100100. 10.1016/j.jlr.2021.100100 34331935 PMC8387743

[45] Dorninger F Brodde A Braverman NE Moser AB Just WW Forss-Petter S Homeostasis of phospholipids — the level of phosphatidylethanolamine tightly adapts to changes in ethanolamine plasmalogens. *Biochim Et Biophys Acta (BBA) Mol Cell Biol Lip.* (2015) 1851:117–28. 10.1016/j.bbalip.2014.11.005 25463479 PMC4331674

[46] Rusiñol AE Cui Z Chen MH Vance JE. A unique mitochondria-associated membrane fraction from rat liver has a high capacity for lipid synthesis and contains pre-golgi secretory proteins including nascent lipoproteins. *J Biol Chem.* (1994) 269:27494–502. 10.1016/s0021-925847012-3 7961664

[47] Voelker DR. The ATP-dependent translocation of phosphatidylserine to the mitochondria is a process that is restricted to the autologous organelle. *J Biol Chem.* (1993) 268:7069–74. 10.1016/s0021-925853146-x 8463240

[48] Wu W-I Voelker DR. Reconstitution of phosphatidylserine transport from chemically defined donor membranes to phosphatidylserine decarboxylase 2 implicates specific lipid domains in the process. *J Biol Chem.* (2004) 279:6635–42. 10.1074/jbc.m311570200 14660568

[49] Lahiri S Chao JT Tavassoli S Wong AK Choudhary V Young BP A conserved endoplasmic reticulum membrane protein complex (EMC) facilitates phospholipid transfer from the ER to mitochondria. *PLoS Biol.* (2014) 12:1001969. 10.1371/journal.pbio.1001969 25313861 PMC4196738

[50] Voelker DR. Disruption of phosphatidylserine translocation to the mitochondria in baby hamster kidney cells. *J Biol Chem.* (1985) 260:14671–6. 10.1016/s0021-925838623-4 2997219

[51] Voelker DR. Phosphatidylserine translocation to the mitochondrion is an ATP-dependent process in permeabilized animal cells. *Proc Natl Acad Sci.USA.* (1989) 86:9921–5. 10.1073/pnas.86.24.9921 2602382 PMC298614

[52] Voelker DR. Reconstitution of phosphatidylserine import into rat liver mitochondria. *J Biol Chem.* (1989) 264:8019–25. 10.1016/s0021-925883144-1 2542259

[53] Trotter PJ Pedretti J Voelker DR. Phosphatidylserine decarboxylase from saccharomyces cerevisiae. isolation of mutants, cloning of the gene, and creation of a null allele. *J Biol Chem.* (1993) 268:21416–24. 10.1016/s0021-925836940-6 8407984

[54] Trotter PJ Pedretti J Yates R Voelker DR. Phosphatidylserine decarboxylase 2 of saccharomyces cerevisiae. *J Biol Chem.* (1995) 270:6071–80. 10.1074/jbc.270.11.6071 7890740

[55] Trotter PJ Voelker DR. Identification of a non-mitochondrial phosphatidylserine decarboxylase activity (PSD2) in the yeast saccharomyces cerevisiae. *J Biol Chem.* (1995) 270:6062–70. 10.1074/jbc.270.11.6062 7890739

[56] Calzada E Onguka O Claypool SM. Phosphatidylethanolamine metabolism in health and disease. *Int Rev Cell Mol Biol.* (2016) 2016:29–88. 10.1016/bs.ircmb.2015.10.001 26811286 PMC4778737

[57] Kainu V Hermansson M Hänninen S Hokynar K Somerharju P. Import of phosphatidylserine to and export of phosphatidylethanolamine molecular species from mitochondria. *Biochim Et Biophys Acta (BBA) Mol Cell Biol Lip.* (2013) 1831:429–37. 10.1016/j.bbalip.2012.11.003 23159415

[58] Tijburg LBM Geelen MJH Van Golde LMG. Biosynthesis of phosphatidylethanolamine via the CDP-ethanolamine route is an important pathway in isolated rat hepatocytes. *Biochem Biophys Res Commun.* (1989) 160:1275–80. 10.1016/s0006-291x80141-x 2499328

[59] Voelker DR. Phosphatidylserine functions as the major precursor of phosphatidylethanolamine in cultured BHK-21 cells. *Proc Natl Acad Sci.* (1984) 81:2669–73. 10.1073/pnas.81.9.2669 6425837 PMC345131

[60] Heise R Fernie AR Stitt M Nikoloski Z. Pool size measurements facilitate the determination of fluxes at branching points in non-stationary metabolic flux analysis: the case of *Arabidopsis thaliana*. *Front Plant Sci.* (2015) 6:386. 10.3389/fpls.2015.00386 26082786 PMC4451360

[61] Nöh K Grönke K Luo B Takors R Oldiges M Wiechert W. Metabolic flux analysis at ultra short time scale: isotopically non-stationary 13C labeling experiments. *J Biotechnol.* (2007) 129:249–67. 10.1016/j.jbiotec.2006.11.015 17207877

[62] Zheng AO Sher A Fridman D Musante CJ Young JD. Pool size measurements improve precision of flux estimates but increase sensitivity to unmodeled reactions outside the core network in isotopically nonstationary metabolic flux analysis (INST-MFA). *Biotechnol J.* (2022) 17:2000427. 10.1002/biot.202000427 35085426

[63] Steenbergen R Nanowski TS Beigneux A Kulinski A Young SG Vance JE. Disruption of the phosphatidylserine decarboxylase gene in mice causes embryonic lethality and mitochondrial defects. *J Biol Chem.* (2005) 280:40032–40. 10.1074/jbc.m506510200 16192276 PMC2888304

[64] Leonardi R Frank MW Jackson PD Rock CO Jackowski S. Elimination of the CDP-ethanolamine pathway disrupts hepatic lipid homeostasis. *J Biol Chem.* (2009) 284:27077–89. 10.1074/jbc.m109.031336 19666474 PMC2785637

[65] Selathurai A Kowalski GM Burch ML Sepulveda P Risis S Lee-Young RS The CDP-ethanolamine pathway regulates skeletal muscle diacylglycerol content and mitochondrial biogenesis without altering insulin sensitivity. *Cell Metab.* (2015) 21:718–30. 10.1016/j.cmet.2015.04.001 25955207

[66] Fullerton MD Hakimuddin F Bakovic M. Developmental and metabolic effects of disruption of the mouse CTP:phosphoethanolamine cytidylyltransferase gene (Pcyt2). *Mol Cell Biol.* (2007) 27:3327–36. 10.1128/MCB.01527-06 17325045 PMC1899976

[67] Shiao Y-J Lupo G Vance JE. Evidence that phosphatidylserine is imported into mitochondria via a mitochondria-associated membrane and that the majority of mitochondrial phosphatidylethanolamine is derived from decarboxylation of phosphatidylserine. *J Biol Chem.* (1995) 270:11190–8. 10.1074/jbc.270.19.11190 7744750

[68] Birner R Bürgermeister M Schneiter R Daum G. Roles of phosphatidylethanolamine and of its several biosynthetic pathways in saccharomyces cerevisiae. *Mol Biol Cell.* (2001) 12:997–1007. 10.1091/mbc.12.4.997 11294902 PMC32282

[69] Bleijerveld OB Brouwers JFHM Vaandrager AB Helms JB Houweling M. The CDP-ethanolamine pathway and phosphatidylserine decarboxylation generate different phosphatidylethanolamine molecular species. *J Biol Chem.* (2007) 282:28362–72. 10.1074/jbc.m703786200 17673461

[70] Kimura AK Kim H-Y. Phosphatidylserine synthase 2: high efficiency for synthesizing phosphatidylserine containing docosahexaenoic acid. *J Lip Res.* (2013) 54:214–22. 10.1194/jlr.m031989 23071296 PMC3520527

[71] Petkevicius K Palmgren H Glover MS Ahnmark A Andréasson A-C Madeyski-Bengtson K TLCD1 and TLCD2 regulate cellular phosphatidylethanolamine composition and promote the progression of non-alcoholic steatohepatitis. *Nat Commun.* (2022) 13:33735. 10.1038/s41467-022-33735-6 36241646 PMC9568529

[72] King MW. *Integrative medical biochemistry examination and board review.* McGraw Hill (2014). Available online at: https://accesspharmacy.mhmedical.com/content.aspx?bookid=1696&sectionid=11139909 (accessed October 20, 2022).

[73] DeLong CJ Shen Y-J Thomas MJ Cui Z. Molecular distinction of phosphatidylcholine synthesis between the CDP-choline pathway and phosphatidylethanolamine methylation pathway. *J Biol Chem.* (1999) 274:29683–8. 10.1074/jbc.274.42.29683 10514439

[74] Vance DE. Phospholipid methylation in mammals: from biochemistry to physiological function. *Biochim Et Biophys Acta (BBA) Biomem.* (2014) 1838:1477–87. 10.1016/j.bbamem.2013.10.018 24184426

[75] Li Z Agellon LB Allen TM Umeda M Jewell L Mason A The ratio of phosphatidylcholine to phosphatidylethanolamine influences membrane integrity and steatohepatitis. *Cell Metab.* (2006) 3:321–31. 10.1016/j.cmet.2006.03.007 16679290

[76] Vance D Vance JE. *Biochemistry of lipids, lipoproteins, and membranes.* 4th ed. Amsterdam: Elsevier (1996).

[77] Leventis PA Grinstein S. The distribution and function of phosphatidylserine in cellular membranes. *Ann Rev Biophys.* (2010) 39:407–27. 10.1146/annurev.biophys.093008.131234 20192774

[78] van der Veen JN Kennelly JP Wan S Vance JE Vance DE Jacobs RL. The critical role of phosphatidylcholine and phosphatidylethanolamine metabolism in health and disease. *Biochim Et Biophys Acta (BBA) Biomemb.* (2017) 1859:15581572. 10.1016/j.bbamem.2017.04.006 28411170

[79] Bremer J Figard PH Greenberg DM. The biosynthesis of choline and its relation to phospholipid metabolism. *Biochim Et Biophys Acta.* (1960) 43:477–88. 10.1016/0006-300290470-4

[80] Kuge O Hasegawa K Ohsawa T Saito K Nishijima M. Purification and characterization of Chinese hamster phosphatidylserine synthase 2. *J Biol Chem.* (2003) 278:42692–8. 10.1074/jbc.m307270200 12912985

[81] Kuge O Nishijima M Akamatsu Y. Isolation of a somatic-cell mutant defective in phosphatidylserine biosynthesis. *Proc Natl Acad Sci USA.* (1985) 82:1926–30. 10.1073/pnas.82.7.1926 3856869 PMC397448

[82] Voelker DR Frazier JL. Isolation and characterization of a Chinese hamster ovary cell line requiring ethanolamine or phosphatidylserine for growth and exhibiting defective phosphatidylserine synthase activity. *J Biol Chem.* (1986) 261:1002–8. 10.1016/s0021-925836044-1 3003047

[83] Vance JE. Thematic review series: glycerolipids. phosphatidylserine and phosphatidylethanolamine in mammalian cells: two metabolically related aminophospholipids. *J Lip Res.* (2008) 49:1377–87. 10.1194/jlr.r700020-jlr200 18204094

[84] Arikketh D Nelson R Vance JE. Defining the importance of phosphatidylserine synthase-1 (PSS1). *J Biol Chem.* (2008) 283:12888–97. 10.1074/jbc.m800714200 18343815

[85] Bergo MO Gavino BJ Steenbergen R Sturbois B Parlow AF Sanan DA Defining the importance of phosphatidylserine synthase 2 in mice. *J Biol Chem.* (2002) 277:47701–8. 10.1074/jbc.m207734200 12361952

[86] Emoto K Kobayashi T Yamaji A Aizawa H Yahara I Inoue K Redistribution of phosphatidylethanolamine at the cleavage furrow of dividing cells during cytokinesis. *Proc Natl Acad Sci USA.* (1996) 93:12867–72. 10.1073/pnas.93.23.12867 8917511 PMC24012

[87] Emoto K Umeda M. An essential role for a membrane lipid in cytokinesis. *J Cell Biol.* (2000) 149:1215–24. 10.1083/jcb.149.6.1215 10851019 PMC2175113

[88] Kreutzberger AJB Kiessling V Liang B Yang S-T Castle JD Tamm LK. Asymmetric phosphatidylethanolamine distribution controls fusion pore lifetime and probability. *Biophys J.* (2017) 113:1912–5. 10.1016/j.bpj.2017.09.014 29037600 PMC5685784

[89] Binotti B Jahn R Pérez-Lara Á. An overview of the synaptic vesicle lipid composition. *Arch Biochem Biophys.* (2021) 709:108966. 10.1016/j.abb.2021.108966 34139199

[90] Takamori S Holt M Stenius K Lemke EA Grønborg M Riedel D Molecular anatomy of a trafficking organelle. *Cell.* (2006) 127:831–46. 10.1016/j.cell.2006.10.030 17110340

[91] Deutsch JW Kelly RB. Lipids of synaptic vesicles: relevance to the mechanism of membrane fusion. *Biochemistry.* (1981) 20:378–85. 10.1021/bi00505a024 7470487

[92] Nagy A Baker RR Morris SJ Whittaker VP. The preparation and characterization of synaptic vesicles of high purity. *Brain Res.* (1976) 109:285–309. 10.1016/0006-899390531-x 132227

[93] Grapentine S Agarwal P Dolinsky V Bakovic M. Phosphoethanolamine reverses aberrant DNA methylation in non-alcoholic steatohepatitis caused by PCYT2 deficiency. *Res Square.* (2022) 41:546–960. 10.21203/rs.3.rs-2143064/v1

[94] van Meer G Voelker DR Feigenson GW. Membrane lipids: where they are and how they behave. *Nat Rev Mol Cell Biol.* (2008) 9:112–24. 10.1038/nrm2330 18216768 PMC2642958

[95] Joubert F Puff N. Mitochondrial cristae architecture and functions: lessons from minimal model systems. *Membranes.* (2021) 11:465. 10.3390/membranes11070465 34201754 PMC8306996

[96] Böttinger L Horvath SE Kleinschroth T Hunte C Daum G Pfanner N Phosphatidylethanolamine and cardiolipin differentially affect the stability of mitochondrial respiratory chain supercomplexes. *J Mol Biol.* (2012) 423:677–86. 10.1016/j.jmb.2012.09.001 22971339 PMC3480645

[97] Calzada E McCaffery JM Claypool SM. Phosphatidylethanolamine made in the inner mitochondrial membrane is essential for yeast cytochrome bc1complex function. *Nat Commun.* (2018) 10:1432. 10.1101/269233 PMC6441012 30926815

[98] Tamura Y Endo T Iijima M Sesaki H. Ups1p and ups2p antagonistically regulate cardiolipin metabolism in mitochondria. *J Cell Biol.* (2009) 185:1029–45. 10.1083/jcb.200812018 19506038 PMC2711612

[99] Osman C Haag M Potting C Rodenfels J Dip PV Wieland FT The genetic interactome of prohibitins: coordinated control of cardiolipin and phosphatidylethanolamine by conserved regulators in mitochondria. *J Cell Biol.* (2009) 184:583–96. 10.1083/jcb.200810189 19221197 PMC2654118

[100] Kuroda T Tani M Moriguchi A Tokunaga S Higuchi T Kitada S FMP30 is required for the maintenance of a normal cardiolipin level and mitochondrial morphology in the absence of mitochondrial phosphatidylethanolamine synthesis. *Mol Microbiol.* (2011) 80:248–65. 10.1111/j.1365-2958.2011.07569.x 21306442

[101] Becker T Horvath SE Böttinger L Gebert N Daum G Pfanner N. Role of phosphatidylethanolamine in the biogenesis of mitochondrial outer membrane proteins. *J Biol Chem.* (2013) 288:16451–9. 10.1074/jbc.m112.442392 23625917 PMC3675581

[102] Dihanich M Suda K Schatz G. A yeast mutant lacking mitochondrial porin is respiratory-deficient, but can recover respiration with simultaneous accumulation of an 86-KD extramitochondrial protein. *EMBO J.* (1987) 6:723–8. 10.1002/j.1460-2075.1987.tb04813.x 2438132 PMC553456

[103] Ellenrieder L Dieterle MP Doan KN Mårtensson CU Floerchinger A Campo ML Dual role of mitochondrial porin in metabolite transport across the outer membrane and protein transfer to the inner membrane. *Mol Cell.* (2019) 73:14. 10.1016/j.molcel.2018.12.014 30738704

[104] Campos JC Bozi LH Ferreira JC. Mitochondrial biogenesis and dynamics in health and disease. *Essent Aspects Immunometab Health Dis.* (2021) 2021:31–51. 10.1007/978-3-030-86684-6_3

[105] Joshi AS Thompson MN Fei N Hüttemann M Greenberg ML. Cardiolipin and mitochondrial phosphatidylethanolamine have overlapping functions in mitochondrial fusion in saccharomyces cerevisiae. *J Biol Chem.* (2012) 287:17589–97. 10.1074/jbc.m111.330167 22433850 PMC3366806

[106] Signorell A Gluenz E Rettig J Schneider A Shaw MK Gull K Perturbation of phosphatidylethanolamine synthesis affects mitochondrial morphology and cell-cycle progression in procyclic-formtrypanosoma brucei. *Mol Microbiol.* (2009) 72:1068–79. 10.1111/j.1365-2958.2009.06713.x 19400804

[107] Okamoto K Shaw JM. Mitochondrial morphology and dynamics in yeast and multicellular eukaryotes. *Ann Rev Genet.* (2005) 39:503–36. 10.1146/annurev.genet.38.072902.093019 16285870

[108] Chan DC. Mitochondrial fusion and fission in mammals. *Ann Rev Cell Dev Biol.* (2006) 22:79–99. 10.1146/annurev.cellbio.22.010305.104638 16704336

[109] Song Z Chen H Fiket M Alexander C Chan DC. OPA1 processing controls mitochondrial fusion and is regulated by mrna splicing, membrane potential, and YME1L. *J Cell Biol.* (2007) 178:749–55. 10.1083/jcb.200704110 17709429 PMC2064540

[110] Wong ED Wagner JA Gorsich SW McCaffery JM Shaw JM Nunnari J. The dynamin-related Gtpase, MGM1P, is an intermembrane space protein required for maintenance of fusion competent mitochondria. *J Cell Biol.* (2000) 151:341–52. 10.1083/jcb.151.2.341 11038181 PMC2192650

[111] Chan EYL McQuibban GA. Phosphatidylserine decarboxylase 1 (PSD1) promotes mitochondrial fusion by regulating the biophysical properties of the mitochondrial membrane and alternative topogenesis of mitochondrial genome maintenance protein 1 (MGM1). *J Biol Chem.* (2012) 287:40131–9. 10.1074/jbc.m112.399428 23045528 PMC3504727

[112] Rockenfeller P Koska M Pietrocola F Minois N Knittelfelder O Sica V Phosphatidylethanolamine positively regulates autophagy and longevity. *Cell Death Different.* (2015) 22:499–508. 10.1038/cdd.2014.219 25571976 PMC4326582

[113] Ichimura Y Kirisako T Takao T Satomi Y Shimonishi Y Ishihara N A ubiquitin-like system mediates protein lipidation. *Nature.* (2000) 408:488–92. 10.1038/35044114 11100732

[114] Bento CF Renna M Ghislat G Puri C Ashkenazi A Vicinanza M Mammalian autophagy: how does it work? *Ann Rev Biochem.* (2016) 85:685–713. 10.1146/annurev-biochem-060815-014556 26865532

[115] Kabeya Y. LC3, a mammalian homologue of yeast APG8P, is localized in autophagosome membranes after processing. *EMBO J.* (2000) 19:5720–8. 10.1093/emboj/19.21.5720 11060023 PMC305793

[116] Tanida I Ueno T Kominami E. LC3 and autophagy. *Methods Mol Biol.* (2008) 445:157. 10.1007/978-1-59745-157-4_4 18425443

[117] Onishi M Yamano K Sato M Matsuda N Okamoto K. Molecular mechanisms and physiological functions of mitophagy. *EMBO J.* (2021) 40:705. 10.15252/embj.2020104705 33438778 PMC7849173

[118] Nguyen TN Padman BS Usher J Oorschot V Ramm G Lazarou M. ATG8 family LC3/GABARAP proteins are crucial for autophagosome–lysosome fusion but not autophagosome formation during Pink1/parkin mitophagy and starvation. *J Cell Biol.* (2016) 215:857–74. 10.1083/jcb.201607039 27864321 PMC5166504

[119] Li J Cao F Yin H-L Huang Z-J Lin Z-T Mao N Ferroptosis: past, present and future. *Cell Death Dis.* (2020) 11:2298. 10.1038/s41419-020-2298-2 32015325 PMC6997353

[120] Dixon SJ Lemberg KM Lamprecht MR Skouta R Zaitsev EM Gleason CE Ferroptosis: an iron-dependent form of nonapoptotic cell death. *Cell.* (2012) 149:1060–72. 10.1016/j.cell.2012.03.042 22632970 PMC3367386

[121] Hanson LR Roeytenberg A Martinez PM Coppes VG Sweet DC Rao RJ Intranasal deferoxamine provides increased brain exposure and significant protection in rat ischemic stroke. *J Pharmacol Exp Ther.* (2009) 330:679–86. 10.1124/jpet.108.149807 19509317 PMC2729791

[122] Raven EP Lu PH Tishler TA Heydari P Bartzokis G. Increased iron levels and decreased tissue integrity in hippocampus of alzheimer’s disease detected in vivo with magnetic resonance imaging. *J Alzheimer’s Dis.* (2013) 37:127–36. 10.3233/jad-130209 23792695

[123] Hambright WS Fonseca RS Chen L Na R Ran Q. Ablation of ferroptosis regulator glutathione peroxidase 4 in forebrain neurons promotes cognitive impairment and neurodegeneration. *Redox Biol.* (2017) 12:8–17. 10.1016/j.redox.2017.01.021 28212525 PMC5312549

[124] Kagan VE Mao G Qu F Angeli JP Doll S Croix CS Oxidized arachidonic and adrenic pes navigate cells to ferroptosis. *Nat Chem Biol.* (2016) 13:81–90. 10.1038/nchembio.2238 27842066 PMC5506843

[125] Luo X Gong H-B Gao H-Y Wu Y-P Sun W-Y Li Z-Q Oxygenated phosphatidylethanolamine navigates phagocytosis of ferroptotic cells by interacting with TLR2. *Cell Death Different.* (2021) 28:1971–89. 10.1038/s41418-020-00719-2 33432112 PMC8185102

[126] Zou Y Henry WS Ricq EL Graham ET Phadnis VV Maretich P Plasticity of ether lipids promotes ferroptosis susceptibility and evasion. *Nature.* (2020) 585:603–8. 10.1038/s41586-020-2732-8 32939090 PMC8051864

[127] Pilch PF Thompson PA Czech MP. Coordinate modulation of D-glucose transport activity and bilayer fluidity in plasma membranes derived from control and insulin-treated adipocytes. *Proc Natl Acad Sci USA.* (1980) 77:915–8. 10.1073/pnas.77.2.915 6987672 PMC348392

[128] Muller S Denet S Candiloros H Barrois R Wiernsperger N Donner M Action of metformin on erythrocyte membrane fluidity in vitro and in vivo. *Eur J Pharmacol.* (1997) 337:103–10. 10.1016/s0014299901287-9 9389387

[129] Dawaliby R Trubbia C Delporte C Noyon C Ruysschaert J-M Van Antwerpen P Phosphatidylethanolamine is a key regulator of membrane fluidity in eukaryotic cells. *J Biol Chem.* (2016) 291:3658–67. 10.1074/jbc.m115.706523 26663081 PMC4751403

[130] Van Der Veen JN Lingrell S McCloskey N Leblond ND Galleguillos D Zhao YY A role for phosphatidylcholine and phosphatidylethanolamine in hepatic insulin signaling. *FASEB J.* (2019) 33:5045–57. 10.1096/fj.201802117r 30615497

[131] Kelly KP Alassaf M Sullivan CE Brent AE Goldberg ZH Poling ME Fat body phospholipid state dictates hunger-driven feeding behavior. *ELife.* (2022) 11:80282. 10.7554/elife.80282 36201241 PMC9566863

[132] Fullerton MD Hakimuddin F Bonen A Bakovic M. The development of a metabolic disease phenotype in CTP:phosphoethanolamine cytidylyltransferase-deficient mice. *J Biol Chem.* (2009) 284:25704–13. 10.1074/jbc.m109.023846 19625253 PMC2757972

[133] Grapentine S Singh RK Basu P Sivanesan S Mattos G Oresajo O PCYT2 deficiency causes age-dependant development of nonalcoholic steatohepatitis and insulin resistance that could be attenuated with phosphoethanolamine. *Sci Rep.* (2022) 12:5140. 10.1038/s41598-022-05140-y 35058529 PMC8776951

[134] Taylor A Schenkel LC Yokich M Bakovic M. Adaptations to excess choline in insulin resistant and pcyt2 deficient skeletal muscle. *Biochem Cell Biol.* (2017) 95:223–31. 10.1139/bcb-2016-0105 28068143

[135] Ahmed MY Al-Khayat A Al-Murshedi F Al-Futaisi A Chioza BA Pedro Fernandez-Murray J A mutation of EPT1 (SELENOI) underlies a new disorder of kennedy pathway phospholipid biosynthesis. *Brain.* (2017) 140:547–54. 10.1093/brain/aww318 28052917 PMC5382949

[136] Girisha KM von Elsner L Neethukrishna K Muranjan M Shukla A Bhavani GSL The homozygous variant c.797g>A/p.(cys266tyr) in pisd is associated with a spondyloepimetaphyseal dysplasia with large epiphyses and disturbed mitochondrial function. *Hum Mutat.* (2018) 40:299–309. 10.1002/humu.23693 30488656

[137] Sousa SB Jenkins D Chanudet E Tasseva G Ishida M Anderson G Gain-of-function mutations in the phosphatidylserine synthase 1 (PTDSS1) gene cause lenz-majewski syndrome. *Nat Genet.* (2013) 46:70–6. 10.1038/ng.2829 24241535

[138] Nerlich A von Orlow M Rontein D Hanson AD Dörmann P. Deficiency in phosphatidylserine decarboxylase activity in the psd1 psd2 psd3 triple mutant of arabidopsis affects phosphatidylethanolamine accumulation in mitochondria. *Plant Physiol.* (2007) 144:904–14. 10.1104/pp.107.095414 17449644 PMC1914192

[139] Tamhankar PM Vasudevan L Bansal V Menon SR Gawde HM D’Souza A Lenz-majewski syndrome: report of a case with novel mutation in PTDSS1 gene. *Eur J Med Genet.* (2015) 58:392–9. 10.1016/j.ejmg.2015.06.002 26117586

[140] Ethanolamine. *FooDB.* (2015). Available online at: https://foodb.ca/compounds/FDB030851#:~:text=Ethanolamine%20can%20be%20found%20in,consumption%20of%20these%20food%20products. (accessed April 7, 2015).

[141] Wang S Zhang S Liou L-C Ren Q Zhang Z Caldwell GA Phosphatidylethanolamine deficiency disrupts α-synuclein homeostasis in yeast and worm models of parkinson disease. *Proc Natl Acad Sci USA.* (2014) 111:4111. 10.1073/pnas.1411694111 25201965 PMC4183298

[142] Ježek J Cooper K Strich R. Reactive oxygen species and mitochondrial dynamics: the yin and yang of mitochondrial dysfunction and cancer progression. *Antioxidants.* (2018) 7:13. 10.3390/antiox7010013 29337889 PMC5789323

[143] Yu T Robotham JL Yoon Y. Increased production of reactive oxygen species in hyperglycemic conditions requires dynamic change of mitochondrial morphology. *Proc Natl Acad Sci USA.* (2006) 103:2653–8. 10.1073/pnas.0511154103 16477035 PMC1413838

[144] Zhao H Wang T. PE homeostasis rebalanced through mitochondria-er lipid exchange prevents retinal degeneration in drosophila. *PLoS Genet.* (2020) 16:1009070. 10.1371/journal.pgen.1009070 33064773 PMC7592913

[145] Saito K Nishijima M Kuge O. Genetic evidence that phosphatidylserine synthase II catalyzes the conversion of phosphatidylethanolamine to phosphatidylserine in Chinese hamster ovary cells. *J Biol Chem.* (1998) 273:17199–205. 10.1074/jbc.273.27.17199 9642289

[146] Yamamoto Y Sakurai T Chen Z Inoue N Chiba H Hui S-P. Lysophosphatidylethanolamine affects lipid accumulation and metabolism in a human liver-derived cell line. *Nutrients.* (2022) 14:579. 10.3390/nu14030579 35276938 PMC8839386

[147] Wilson-Ashworth HA Judd AM Law RM Freestone BD Taylor S Mizukawa MK Formation of transient non-protein calcium pores by *Lysophospholipids* in S49 lymphoma cells. *J Memb Biol.* (2004) 200:25–33. 10.1007/s00232-004-0691-x 15386157

[148] Zampiga M Meluzzi A Sirri F. Effect of dietary supplementation of lysophospholipids on productive performance, nutrient digestibility and carcass quality traits of broiler chickens. *Italian J Animal Sci.* (2016) 15:521–8. 10.1080/1828051x.2016.1192965

[149] Boontiam W Hyun YK Jung B Kim YY. Effects of lysophospholipid supplementation to reduced energy, crude protein, and amino acid diets on growth performance, nutrient digestibility, and blood profiles in broiler chickens. *Poultry Sci.* (2019) 98:6693–701. 10.3382/ps/pex005 31801309 PMC6869753

[150] Huo Q Li B Cheng L Wu T You P Shen S Dietary supplementation of *Lysophospholipids* affects feed digestion in lambs. *Animals.* (2019) 9:805. 10.3390/ani9100805 31618894 PMC6826496

[151] Riekhof WR Wu J Jones JL Voelker DR. Identification and characterization of the major lysophosphatidylethanolamine acyltransferase in saccharomyces cerevisiae. *J Biol Chem.* (2007) 282:28344–52. 10.1074/jbc.m705256200 17652094

[152] Quehenberger O Armando AM Brown AH Milne SB Myers DS Merrill AH Lipidomics reveals a remarkable diversity of lipids in human plasma. *J Lip Res.* (2010) 51:3299–305. 10.1194/jlr.m009449 20671299 PMC2952570

[153] Hung M-C Shibasaki K Yoshida R Sato M Imaizumi K. Learning behaviour and cerebral protein kinase C, antioxidant status, lipid composition in senescence-accelerated mouse: influence of a phosphatidylcholine–vitamin B12diet. *Br J Nutr.* (2001) 86:163–71. 10.1079/bjn2001391 11502229

[154] Cenacchi T Bertoldin T Farina C Fiori MG Crepaldi G. Cognitive decline in the elderly: a double-blind, placebo-controlled multicenter study on efficacy of phosphatidylserine administration. *Aging Clin Exp Res.* (1993) 5:123–33. 10.1007/BF03324139 8323999

[155] Engel RR Satzger W Günther W Kathmann N Bove D Gerke S Double-blind cross-over study of phosphatidylserine vs. placebo in patients with early dementia of the alzheimer type. *Eur Neuropsychopharmacol.* (1992) 2:149–55. 10.1016/0924-977x90025-4 1633433

[156] Delwaide PJ Gyselynck-Mambourg AM Hurlet A Ylieff M. Double-blind randomized controlled study of phosphatidylserine in senile demented patients. *Acta Neurol Scand.* (2009) 73:136–40. 10.1111/j.1600-0404.1986.tb03254.x 3518329

[157] Heiss W-D Kessler J Mielke R Szelies B Herholz K. Long-term effects of phosphatidylserine, pyritinol, and cognitive training in alzheimer’s disease. *Dem Geriatr Cogn Dis.* (1994) 5:88–98. 10.1159/000106702 8038871

[158] Moré MI Freitas U Rutenberg D. Positive effects of soy lecithin-derived phosphatidylserine plus phosphatidic acid on memory, cognition, daily functioning, and mood in elderly patients with alzheimer’s disease and dementia. *Adv Ther.* (2014) 31:1247–62. 10.1007/s12325-014-0165-1 25414047 PMC4271139

[159] Zhao Y-C Zhou M-M Zhang L-Y Cong P-X Xu J Xue C-H Recovery of brain dha-containing phosphatidylserine and ethanolamine plasmalogen after dietary dha-enriched phosphatidylcholine and phosphatidylserine in samp8 mice fed with high-fat diet. *Lip Health Dis.* (2020) 19:1253. 10.1186/s12944-020-01253-3 32450867 PMC7249346

[160] Ikuo I Katsumi I Michihiro S. Absorption and transport of base moieties of phosphatidylcholine and phosphatidylethanolamine in rats. *Biochim Et Biophys Acta (BBA) Lip Lip Metab.* (1987) 921:245–53. 10.1016/0005-276090024-5 3651485

[161] Yamashita S Hashimoto M Haque AM Nakagawa K Kinoshita M Shido O Oral administration of ethanolamine glycerophospholipid containing a high level of plasmalogen improves memory impairment in amyloid β-infused rats. *Lipids.* (2017) 52:575–85. 10.1007/s11745-017-4260-3 28551706

[162] Che H Li Q Zhang T Ding L Zhang L Shi H A comparative study of EPA-enriched ethanolamine plasmalogen and EPA-enriched phosphatidylethanolamine on AB 42 induced cognitive deficiency in a rat model of alzheimer’s disease. *Food Funct.* (2018) 9:3008–17. 10.1039/c8fo00643a 29774334

[163] Fujino T Yamada T Asada T Tsuboi Y Wakana C Mawatari S Efficacy and blood plasmalogen changes by oral administration of plasmalogen in patients with mild alzheimer’s disease and mild cognitive impairment: a multicenter, randomized, double-blind, placebo-controlled trial. *EBio Med.* (2017) 17:199–205. 10.1016/j.ebiom.2017.02.012 28259590 PMC5360580

[164] Dorninger F Forss-Petter S Wimmer I Berger J. Plasmalogens, platelet-activating factor and beyond – ether lipids in signaling and neurodegeneration. *Neurobiol Dis.* (2020) 145:105061. 10.1016/j.nbd.2020.105061 32861763 PMC7116601

[165] Takahashi T Kamiyoshihara R Otoki Y Ito J Kato S Suzuki T Structural changes of ethanolamine plasmalogen during intestinal absorption. *Food Funct.* (2020) 11:8068–76. 10.1039/d0fo01666g 32852024

[166] Kawashima Y Mizuguchi H Musoh K Kozuka H. The mechanism for the increased supply of phosphatidylcholine for the proliferation of biological membranes by clofibric acid, a peroxisome proliferator. *Biochim Et Biophys Acta (BBA) Lip Lip Metab.* (1994) 1212:311–8. 10.1016/0005-276090205-4 8199202

[167] Mizuguchi H Kawashima Y. Alterations by clofibric acid of metabolism of phosphatidylethanolamme in rat-liver. *Biol Pharmaceut Bull.* (1996) 19:1115–20. 10.1248/bpb.19.1115 8889026

[168] Mizuguchi H Kudo N Ohya T Kawashima Y. Effects of tiadenol and di-(2-ethylhexyl)phthalate on the metabolism of phosphatidylcholine and phosphatidylethanolamine in the liver of rats. *Biochem Pharmacol.* (1999) 57:869–76. 10.1016/s0006-295200365-7 10086319

[169] Pritchard PH Chiang PK Cantoni GL Vance DE. Inhibition of phosphatidylethanolamine N-methylation by 3-deazaadenosine stimulates the synthesis of phosphatidylcholine via the CDP-choline pathway. *J Biol Chem.* (1982) 257:6362–7. 10.1016/s0021-925865149-3 7076675

[170] Hess R Stabuli W Riess W. Nature of the hepatomegalic effect produced by ethyl-chlorophenoxy-isobutyrate in the rat. *Nature.* (1965) 208:856–8. 10.1038/208856a0 5870099

[171] Svoboda DJ Azarnoff DL. Response of hepatic microbodies to a hypolipidemic agent. Ethyl chlorophenoxyisobutyrate (CPIB). *J Cell Biol.* (1966) 30:442–50. 10.1083/jcb.30.2.442 5968981 PMC2107000

[172] Lipsky NG Pedersen PL. Perturbation by clofibrate of mitochondrial levels in animal cells. implications for a model of mitochondrial Genesis. *J Biol Chem.* (1982) 257:1473–81. 10.1016/s0021-925868218-9 7056728

[173] Kaneko A Sakamoto S Morita M Onoé T. Morphological and biochemical changes in rat liver during the early stages of ethyl chlorophenoxyisobutyrate administration. *Tohoku J Exp Med.* (1969) 99:81–101. 10.1620/tjem.99.81 4390771

[174] Walkey CJ Donohue LR Bronson R Agellon LB Vance DE. Disruption of the murine gene encoding phosphatidylethanolamine n -methyltransferase. *Proc Natl Acad Sci USA.* (1997) 94:12880–5. 10.1073/pnas.94.24.12880 9371769 PMC24232

[175] Walkey CJ Yu L Agellon LB Vance DE. Biochemical and evolutionary significance of phospholipid methylation. *J Biol Chem.* (1998) 273:27043–6. 10.1074/jbc.273.42.27043 9765216

[176] Zhu X Song J Mar M-H Edwards LJ Zeisel SH. Phosphatidylethanolamine N-methyltransferase (PEMT) knockout mice have hepatic steatosis and abnormal hepatic choline metabolite concentrations despite ingesting a recommended dietary intake of choline. *Biochem J.* (2003) 370:987–93. 10.1042/bj20021523 12466019 PMC1223223

[177] Biserni A Giannessi F Sciarroni AF Milazzo FM Maggi A Ciana P. In vivo imaging reveals selective peroxisome proliferator activated receptor modulator activity of the synthetic ligand 3-(1-(4-chlorobenzyl)-3-t-butylthio-5-isopropylindol-2- yl)-2,2-dimethylpropanoic acid (MK-886). *Mol Pharmacol.* (2008) 73:1434–43. 10.1124/mol.107.042689 18292206

[178] Inestrosa NC Carvajal FJ Zolezzi JM Tapia-Rojas C Serrano F Karmelic D Peroxisome proliferators reduce spatial memory impairment, synaptic failure, and neurodegeneration in brains of a double transgenic mice model of alzheimer’s disease. *J Alzheimer’s Dis.* (2013) 33:941–59. 10.3233/jad-2012-120397 23109558

[179] Blusztajn JK Zeisel SH Wurtman RJ. Synthesis of lecithin (phosphatidylcholine) from phosphatidylethanolamine in bovine brain. *Brain Res.* (1979) 179:319–27. 10.1016/0006-899390447-5 509240

[180] Johnson PI Blusztajn JK. Sexually dimorphic activation of liver and brain phosphatidylethanolamine N-methyltransferase by dietary choline deficiency. *Neurochem Res.* (1998) 23:583–7. 10.1023/a:1022470301550 9566595

[181] Blusztajn JK Zeisel SH Wurtman RJ. Developmental changes in the activity of phosphatidylethanolamine n-methyltransferases in rat brain. *Biochem J.* (1985) 232:505–11. 10.1042/bj2320505 4091805 PMC1152909

[182] Zhu X Mar M-H Song J Zeisel SH. Deletion of the PEMT gene increases progenitor cell mitosis, DNA and protein methylation and decreases calretinin expression in embryonic day 17 mouse hippocampus. *Dev Brain Res.* (2004) 149:121–9. 10.1016/j.devbrainres.2004.01.004 15063092

[183] Cui Z Vance DE. Expression of phosphatidylethanolamine N-methyltransferase-2 is markedly enhanced in long term choline-deficient rats. *J Biol Chem.* (1996) 271:2839–43. 10.1074/jbc.271.5.2839 8576263

[184] Tissue expression of Pemt. *Summary - the human protein atlas.* (2022). Available online at: https://www.proteinatlas.org/ENSG00000133027-PEMT/tissue (accessed December 12, 2022).

